# Reduced dopant-induced scattering in remote charge-transfer-doped MoS_2_ field-effect transistors

**DOI:** 10.1126/sciadv.abn3181

**Published:** 2022-09-21

**Authors:** Juntae Jang, Jae-Keun Kim, Jiwon Shin, Jaeyoung Kim, Kyeong-Yoon Baek, Jaehyoung Park, Seungmin Park, Young Duck Kim, Stuart S. P. Parkin, Keehoon Kang, Kyungjune Cho, Takhee Lee

**Affiliations:** ^1^ Department of Physics and Astronomy, Seoul National University, Seoul 08826, Korea.; ^2^ Max-Planck Institute of Microstructure Physics, Weinberg 2, 06120 Halle, Saale, Germany.; ^3^ Department of Physics, Kyung Hee University, Seoul 02447, Korea.; ^4^ Department of Materials Science and Engineering, Research Institute of Advanced Materials, Seoul National University, Seoul 08826, Korea.; ^5^ Institute of Applied Physics, Seoul National University, Seoul 08826, Korea.; ^6^ Soft Hybrid Materials Research Center, Korea Institute of Science and Technology, Seoul 02792, Korea.

## Abstract

Efficient doping for modulating electrical properties of two-dimensional (2D) transition metal dichalcogenide (TMDC) semiconductors is essential for meeting the versatile requirements for future electronic and optoelectronic devices. Because doping of semiconductors, including TMDCs, typically involves generation of charged dopants that hinder charge transport, tackling Coulomb scattering induced by the externally introduced dopants remains a key challenge in achieving ultrahigh mobility 2D semiconductor systems. In this study, we demonstrated remote charge transfer doping by simply inserting a hexagonal boron nitride layer between MoS_2_ and solution-deposited n-type dopants, benzyl viologen. A quantitative analysis of temperature-dependent charge transport in remotely doped devices supports an effective suppression of the dopant-induced scattering relative to the conventional direct doping method. Our mechanistic investigation of the remote doping method promotes the charge transfer strategy as a promising method for material-level tailoring of electrical and optoelectronic devices based on TMDCs.

## Teaser

Remote charge transfer doping minimizes dopant-induced scattering in two-dimensional transition metal dichalcogenide (TMDC).

## INTRODUCTION

Transition metal dichalcogenides (TMDCs), representative two-dimensional (2D) van der Waals (vdW) semiconducting materials, have gained substantial attention because of their attractive material properties, including their ultrathin 2D nature with superior electronic and optoelectronic properties, making them a strong contender for emerging electronics applications (*1*–*11*). In particular, MoS_2_, an n-type TMDC semiconductor, has been intensively studied in various research fields because it has shown a wide variety of material properties, not only outstanding MoS_2_-based device performances but also strong spin-orbit coupling and valleytronic properties, as well as superconducting properties (*12*–*19*).

To meet the versatile requirements of future electronic and optoelectronic device applications, the electrical properties of TMDCs should ideally be controllable over a wide range on a material level. Doping has been a key element in achieving such control over carrier density, as well as conductivity, and various doping strategies have been developed for expanding the doping range of TMDCs (*20*–*25*). In particular, surface charge transfer doping (SCTD), which involves charge transfer across the interface between TMDCs and externally introduced dopants on the surface, has been intensively studied as an effective method for modulating the electrical properties of TMDCs in a facile manner (*20*). TMDCs, because of their intrinsic 2D nature, can provide a large surface-area-to-volume ratio for highly efficient and sensitive surface charge transfer with dopants adsorbed on the surface. Furthermore, the advantages of SCTD in TMDCs can be amplified by using molecular dopants that can be designed with a high degree of freedom to target dopants with various frontier orbitals and structures for effectively controlling both the carrier type and doping strength (*26*–*28*). Furthermore, the molecular SCTD method in TMDCs occurs via physical adsorption and is believed to have relative advantages in terms of nondestructive and reversible nature over substitutional doping method that involves either a compositional modification during the synthesis (*29*–*31*) or an ion implantation technique (*32*, *33*), both of which accompany structural damages in 2D materials to some extent. An ideal doping method should not only be structurally nondestructive but also be noninvasive in terms of the resulting charge transport (*34*–*36*). Over the past, the concept of spatially separating charged dopants from the conduction channel has been well established as a modulation-doping method (*37*, *38*). This strategy for minimizing dopant-induced scattering effects has realized 2D electron gas in ultrahigh mobility semiconductor systems and has led to the exploration of rich quantum phenomena in 2D, such as quantum Hall effect (*38*, *39*).

Recently, the concept of remote modulation doping in TMDCs has been proposed theoretically by Wang *et al.* (*34*) and demonstrated experimentally by Lee *et al.* (*35*) in a MoS_2_/h-BN/WSe_2_ heterostructure where the doped WSe_2_ acted as the remote source of charges for realizing doping in MoS_2_ while suppressing Coulomb scattering by the charged dopants. However, a precise mechanistic understanding of such remote doping strategy and the resulting enhanced charge transport is required for fully using the promising doping method in TMDCs. Accordingly, our recent study on the dopant-induced scattering effects in the charge transport of WSe_2_ field-effect transistors (FETs) doped with molecular SCTD can provide a route for gaining additional insights into the remote charge transfer and charged impurity scattering processes (*40*). In this study, we extend our analytical framework to investigate the remote charge transfer doping method quantitatively. We demonstrate that placing only a thin h-BN layer between a MoS_2_ conduction channel and solution-deposited molecular dopants is sufficient (i.e., without the WSe_2_ for providing the band offsets) for achieving remote charge transfer in MoS_2_, which provides a simple testbed for systematically studying the suppression of charged impurity scattering via temperature-dependent four-point probe transport measurements. As a result, this noninvasive remote doping method was shown to achieve notably higher channel mobility relative to the conventional direct doping method because of a substantially reduced effective-charged impurity density, which is supported by our modified scattering model, as well as a more reliable doping controllability relative to the conventional direct doping method.

## RESULTS

### h-BN/MoS_2_ vdW heterostructure devices for remote charge transfer doping

**Fig. 1.**
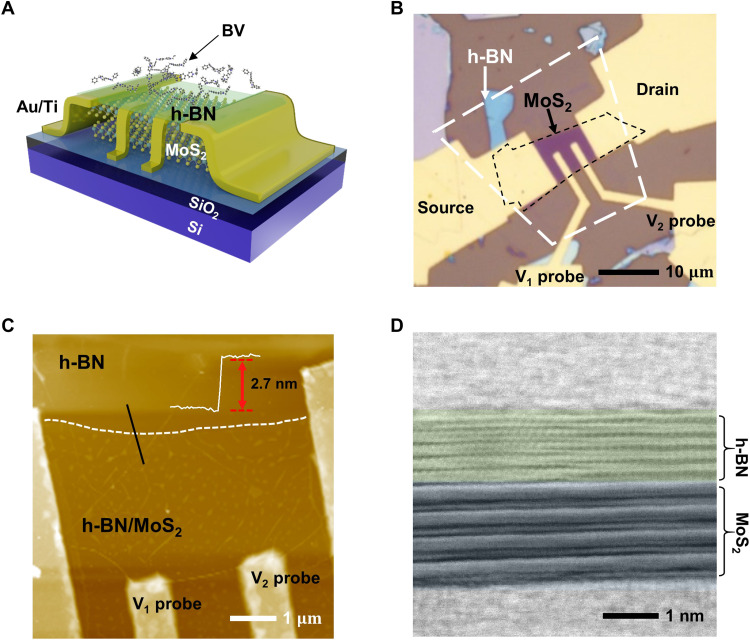
h-BN/MoS_2_ vdW heterostructure devices for remote charge transfer doping. (**A**) The schematic image of BV-doped h-BN/MoS_2_ FET with Au/Ti metal contacts for four-point probe measurements. (**B**) Optical image of h-BN/MoS_2_ FET before BV doping. (**C**) AFM image of h-BN/MoS_2_ FET. The inset shows topographic height profile along the black line, indicating the thickness of the MoS_2_ channel of ~2.7 nm. (**D**) Cs-corrected STEM image of the h-BN/MoS_2_ heterostructure consisting of five layers of h-BN and four layers of MoS_2_.

In this study, our system of interest consists of a MoS_2_ channel doped with benzyl viologen (BV) molecules that are spatially separated by an h-BN layer. To demonstrate doping effects in such a system experimentally, we fabricated MoS_2_ FET devices with metal contacts for four-point probe measurement, followed by the vdW dry transfer of a thin h-BN layer on the MoS_2_ channel area (the fabrication details are provided in Materials and Methods and fig. S1). The device structure of the remotely BV-doped MoS_2_ FETs is shown schematically in Fig. 1A. The optical image of a fabricated device before BV deposition is shown in Fig. 1B. An atomic force microscope (AFM) was used to check the surface morphology after a thin h-BN flake was transferred on top of the MoS_2_ FET. We could barely observe any blisters or wrinkles in the AFM image (Fig. 1C), indicating that the h-BN was properly transferred and the heterostructure was well formed. The thickness of the MoS_2_ channel flake was determined from the AFM image as ~2.7 nm (inset in Fig. 1C and detailed information in fig. S2). The cross-sectional spherical aberration-corrected scanning transmission electron microscopy (Cs-corrected STEM) image of a heterostructure, which confirms the well-stacked h-BN/MoS_2_ heterostructures of this particular sample, consisting of five layers of h-BN (~2 nm thick) and four-layers of MoS_2_ (~3 nm thick) is shown in Fig. 1D (see fig. S3 for representative device samples). To demonstrate remote charge transfer doping, we specifically selected uniform, large, and especially thin h-BN flakes among the mechanically exfoliated h-BN flakes to cover devices because the thickness of the inserted h-BN layer is a critical factor in determining the remote charge transfer doping efficiency of molecular dopants; the thicker the h-BN layer, the lower the expected doping efficiency. The few-layer MoS_2_ channel was selected in this study because of its high surface area–to–volume ratio, which is ideal for investigating the influence of surface-charged impurities generated by the molecular dopants on the charge transport in the remotely doped MoS_2_ FETs (*41*).

### Charge transfer doping of BV molecules in h-BN/MoS_2_ FETs

**Fig. 2.**
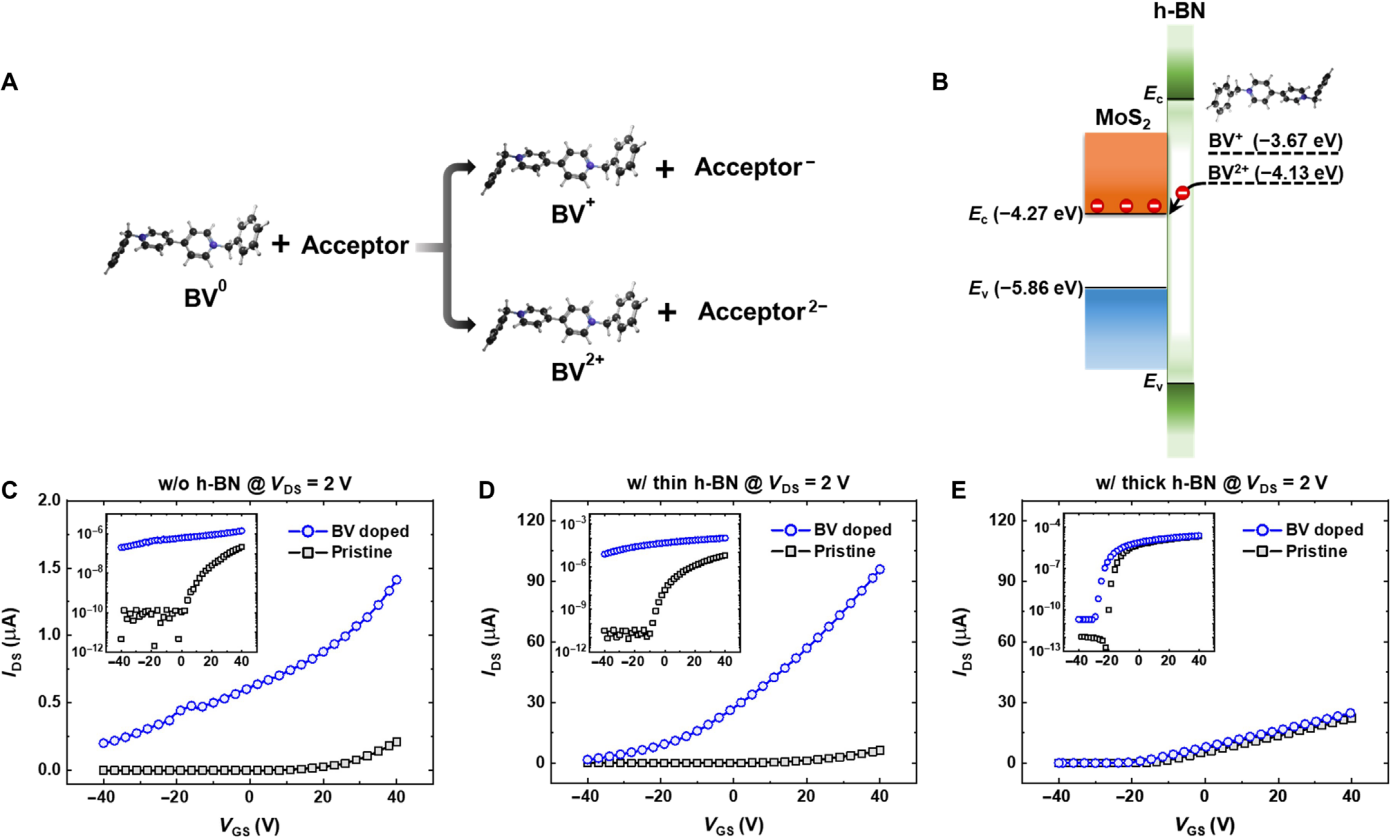
Charge transfer doping of BV molecules in h-BN/MoS_2_ FETs. (**A**) Molecular structure of BV dopant and two different charge transfer doping processes in BV molecules and acceptors (MoS_2_). (**B**) HOMO levels of the two redox states of BV molecule and the energy levels of h-BN and MoS_2_. (**C** to **E**) *I*_DS_-*V*_GS_ curve of MoS_2_ FETs (C) without h-BN and with (D) a thin h-BN (~2 nm) or (E) a thick h-BN layer (>~30 nm), measured at *V*_DS_ = 2 V before BV treatment (pristine, black squares) and after BV treatment (BV doped, blue circles). The logarithmic scale curves are provided in the insets.

BV molecule is a strong reductant that has been widely used for SCTD in various nanomaterials as an n-type dopant (*20*, *42*). The molecular structure of the BV molecule and two different charge transfer processes are available for BV molecules (Fig. 2A). As an effective donor, a neutral BV molecule (BV^0^) can transfer an electron to an acceptor (in this study, MoS_2_), forming a BV^+^ cation, which can then be further oxidized to BV^2+^ by transferring another electron to MoS_2_. The energy levels of the two redox states of a BV molecule and the corresponding energy levels of h-BN and MoS_2_ are illustrated in Fig. 2B. Because the highest occupied molecular orbital (HOMO) of BV is located above the conduction band minimum of MoS_2_, it serves as an electron donor (*43*, *44*). We anticipate that charge transfer can occur from molecular dopants to the MoS_2_ channel through insulating the h-BN interlayer, provided that the h-BN interlayer is thin enough for sufficient tunneling (Fig. 2B). This remote charge transfer doping strategy enables us to successfully modulate the electrical properties of MoS_2_ FETs while minimizing scattering induced by the surface charge dopants.

To confirm the aforementioned remote charge transfer doping in the MoS_2_ channel through a thin h-BN flake by BV molecular dopants, we compared the doping effects of direct doping (i.e., dopants deposited directly on the surface of a bare MoS_2_ channel) and remote doping (dopants separated from MoS_2_ channel by a thin h-BN interlayer) by preparing three different types of MoS_2_ FETs: (i) without h-BN encapsulation, (ii) with a thin h-BN layer (<~2 nm), and (iii) with a thick h-BN layer (>~30 nm). Figure 2 (C to E) shows the transfer curves (source-drain current versus gate voltage; *I*_DS_-*V*_GS_) of MoS_2_ FETs without h-BN (Fig. 2C), with a thin h-BN layer (Fig. 2D), and with a thick h-BN layer (Fig. 2E) before BV treatment (pristine) and after BV treatment (BV doped). The transfer curves on the logarithmic scale are provided in the insets of Fig. 2 (C to E). Without the h-BN layer, the transfer curve after direct doping in Fig. 2C shows an increase in current and the shift of the threshold voltages to the negative gate voltage direction, corresponding to the n-doping of MoS_2_ (*20*). We could confirm a notable doping effect in the remote charge transfer doping case shown in Fig. 2D, observing a current increase and a similar shift of the threshold voltage to a negative gate voltage direction despite inserting a thin h-BN layer (<~2 nm) between the MoS_2_ channel and BV molecules, and spatially separating them from each other. This can be considered analogous to enhanced charge injection from the insertion of a thin h-BN layer between MoS_2_ and metal electrodes via tunneling injection rather than hindering the charge injection owing to the insulating nature of the h-BN layer (*45*, *46*). To exclude the possibility that the thin h-BN layer does not encapsulate the device properly so that the BV molecules may directly dope the MoS_2_, we examined the electrical hysteresis of the MoS_2_ FETs with and without the h-BN layer under ambient conditions. We observed a substantially smaller hysteresis window in the device encapsulated with a thin h-BN flake compared to a bare MoS_2_ device (i.e., without the h-BN encapsulation) and confirmed that even a thin h-BN layer could provide enough encapsulation effect (see fig. S4) (*11*). In contrast to the effective charge transfer doping observed for the thin h-BN interlayer, the MoS_2_ FET covered with a thick h-BN (>~30 nm; see fig. S5 for more details) demonstrated a noticeably low doping efficiency (Fig. 2E), supporting the critical role of the h-BN thin thickness in achieving substantial doping effects via the remote charge transfer. The thick h-BN interlayer is expected to limit the amount of charge transfer from the BV dopant to the underlying MoS_2_ layer. Thus, we confirmed that the exponential decay of Δ*n* (i.e., the doping effect) with increasing thickness of the h-BN interlayer is expected from the decreasing charge tunneling probability through the h-BN interlayer (see fig. S6 for more details). In addition, regarding the stability of the remote doping method, we also characterized the doping stability of the remotely doped devices over time. We confirmed that the remotely doped devices were highly stable under ambient conditions and the remote doping effect in the transfer characteristics were still preserved over time, up to 6 weeks of air exposure (see fig. S7 for more details).

### Doping controllability of remote charge transfer in h-BN/MoS_2_ FETs

To apply the remote charge transfer strategy for emerging electronic applications, both doping controllability and available doping range have to be characterized to tailor the electrical properties of the devices by changing dopant density. Therefore, we measured the electrical properties of remotely doped MoS_2_ FETs and compared them with the results from un-encapsulated devices by changing the concentration of BV solution from 1 to 10 mM. In particular, we characterized the devices at 10 K in a vacuum to eliminate the contributions of phonon scattering and external defects from the environment toward the overall charge transport.

**Fig. 3.**
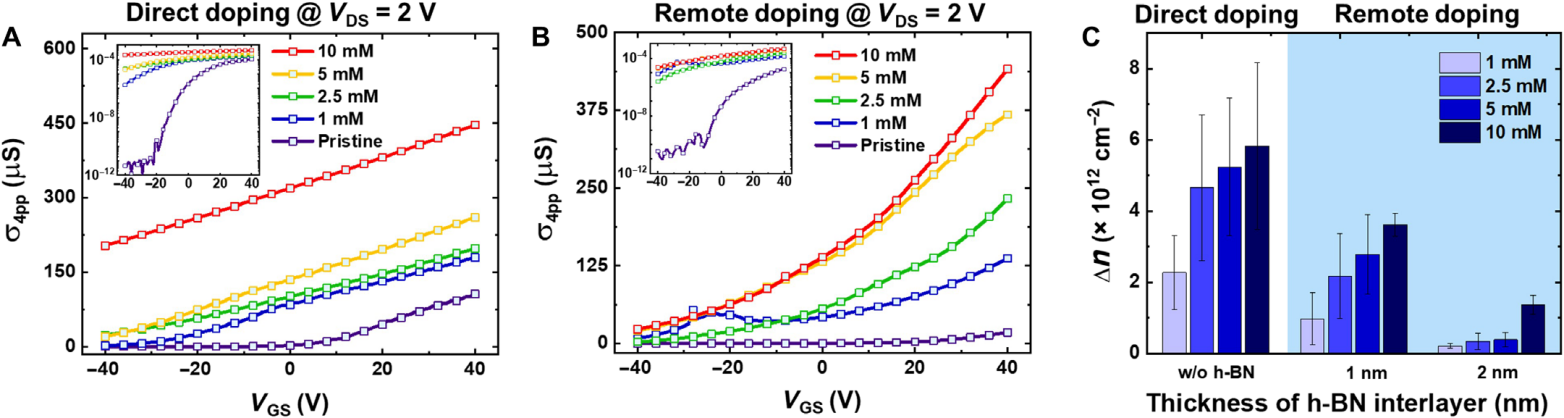
Doping controllability of remote charge transfer doping in h-BN/MoS_2_ FETs. σ_4pp_ versus *V*_GS_ curves for (**A**) directly doped (without h-BN) devices and (**B**) remotely doped (with thin h-BN) devices with different concentrations of BV solution from 1 to 10 mM at 10 K. The logarithmic scale curves are provided in the insets. (**C**) The amount of increased carrier density (∆*n*) by doping for different concentrations of BV solution from 1 to 10 mM at 10 K. The left column set represents ∆*n* in directly doped devices. The middle and the right column sets represent ∆*n* in remotely doped devices with a 1-nm-thick and a 2-nm-thick h-BN interlayer, respectively.

Because the contact resistance of MoS_2_ FETs decreases with BV doping (*20*), we conducted gated four-point probe measurements to focus on the intrinsic charge transport properties of the MoS_2_ channel by minimizing the effect of the varying charge injection behavior because of the BV doping (see fig. S8 for more details). Note that the four-point probe measurement analysis required an offset correction because of the measurement setup with limited voltage resolution (see fig. S9 in section S9 for more details). Figure 3 shows the four-point probe conductance (σ_4pp_) versus gate voltage curves for directly doped (without h-BN) devices (Fig. 3A) and remotely doped (with thin h-BN) devices (Fig. 3B). The measurements were performed on undoped devices (denoted as “pristine”) and doped devices with various concentrations of BV solution from 1 to 10 mM. The conductance curves in the logarithmic scale are provided in the insets of Fig. 3 (A and B). As the doping concentration increased in directly doped devices, σ_4pp_ increased and the curves shifted to the negative gate voltage direction, indicating an increase in the degree of n-doping (Fig. 3A). Note that because of the elimination of the contact resistance effects in four-point probe measurements (see fig. S10A), the σ_4pp_ values under the same doping conditions (i.e., same concentration), were higher than the two-point probe conductance (σ_2pp_) values.

Critically, we repeated the measurements with the remotely doped MoS_2_ FETs through a thin h-BN layer, which revealed a similar enhancement in conductance as the doping concentration increased (Fig. 3B). With these results, we can successfully validate the controllability of remote charge transfer doping, which allows us to tailor the electrical properties by varying the dopant solution concentration. Furthermore, our FET characteristics point to clear advantages of remote doping in terms of suppressing dopant-induced scattering effects. The improved σ_4pp_ values that can be observed with remotely doped devices (Fig. 3B) relative to their pristine state could be a sign of a substantially improved mobility of the remotely doped MoS_2_ channel compared to that of the directly doped device (Fig. 3A). The difference becomes clear when normalized σ_4pp_ plots of the directly doped and remotely doped devices are used (see fig. S11 for more details). The detailed analysis related to the underlying dopant-induced scattering effect will be discussed in the next sections.

To compare the doping strength of direct and remote doping, we extracted the amount of increased carrier density by doping (Δ*n*) using the equation Δ*n* = (*C*_i_ × Δ*V*_th_)/*q*, where *C*_i_ is the capacitance per unit area of the SiO_2_ layer (*C*_i_ = 1.3 × 10^−4^ F/m^2^), $\Delta V_{\text{th}}\;(=\;V_{\text{th}}^{\text{doped}}-V_{\text{th}}^{\text{pristine}})$ is the threshold voltage shift of doped MoS_2_ devices relative to the pristine devices, and *q* is the elementary charge. The obtained Δ*n* values of directly and remotely doped MoS_2_ devices are summarized in Fig. 3C. We sorted out the results by h-BN interlayer thickness to investigate the effect of spatial separation between molecular dopants and MoS_2_ channels. Note that the largest controllable range of ∆*n* for direct doping (5.83 × 10^12^ cm^−2^) was notably higher than that for remote doping (3.62 × 10^12^ cm^−2^ for 1-nm h-BN and 1.36 × 10^12^ cm^−2^ for 2-nm h-BN), implying that the amount of charge transfer decreased in remote doping because of spatial separation. Here, we extracted the largest carrier concentration after doping values, *n*_final_ values, by using *n*_final_ = *C*_i_ (*V*_GS_ − $V_{\text{th}}^{\text{doped}}$)/*q* at *V*_GS_ = 80 V. It was found that the *n*_final_ in directly doped devices with 10 mM concentration of BV solution is determined to be 9.02 × 10^12^ cm^−2^. In remotely doped devices with 10 mM concentration of BV, the *n*_final_ is determined to be 6.46 × 10^12^ cm^−2^ and 3.32 × 10^12^ cm^−2^ for the devices with 1- and 2-nm h-BN remotely doped devices, respectively (see fig. S12). This result is supported by the smaller controllable range of Δ*n* for the 2-nm h-BN case compared to the 1-nm h-BN case. Overall, the larger spatial separation leads to the smaller amount of charge transfer. The reduced dopant-induced scattering can be expected because of the reduced amount of charge transfer by spatial separation. We will discuss the dopant-induced scattering effect in a later section.

### Identifying dopant-induced scattering effects from temperature-dependent mobility measurements

With regard to the mobility of TMDC materials, the mobility can show both increasing and decreasing tendency with carrier density in different carrier density regimes, which originated from various factors, including the screening effect and thickness of TMDCs (*47*–*50*). However, in a well-controlled system that can neglect other factors except for the carrier concentration, Cui *et al.* (*48*) reported that the increased mobility of MoS_2_ as carrier density increases because of the enhanced screening of charged impurity potential. It is rational to assume that electrostatic doping by field effect does not introduce additional impurity scattering, whereas the adsorbed dopant molecules on the TMDC surface can act as an additional charged impurity source in SCTD. To effectively control the conductivity of TMDCs via SCTD, it is essential to minimize additional charged impurity scattering that could be introduced by the SCTD. Because remote doping creates a spatial separation between an electronic channel and charged dopants, it can reduce the Coulomb potential from the dopants, resulting in reduced charged impurity scattering. Therefore, the remote doping method can be a suitable candidate for efficiently and reliably controlling the electrical properties of TMDCs.

**Fig. 4.**
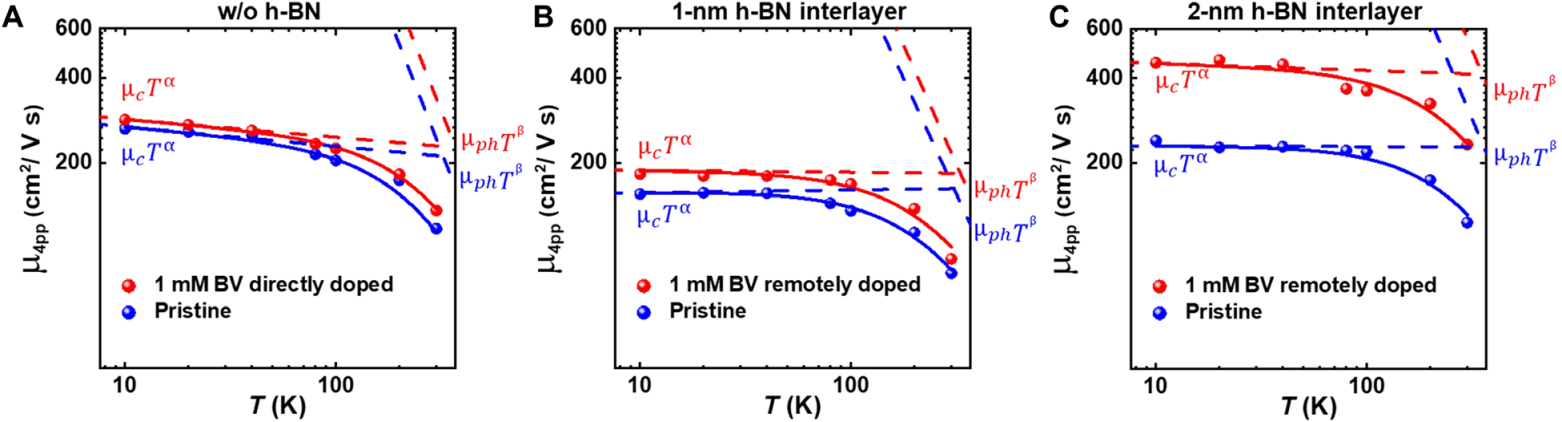
Temperature-dependent mobility measurements. Temperature-dependent mobility values of (**A**) directly doped, (**B**) remotely doped devices with a 1-nm h-BN interlayer, and (**C**) remotely doped devices with a 2-nm h-BN interlayer at temperatures from 10 to 300 K. The mobility values were extracted from the four-point probe conductance plots at *V*_GS_ = 80 V for pristine devices (blue lines) and doped devices (red lines) and under similar Δ*n* conditions.

To investigate the effects of additional introduced charged impurities by adsorbed charge transfer dopants on the surface, the effect of phonon scattering should be excluded by lowering the temperature because charged impurity scattering and phonon scattering are the most dominant sources of charge transport hindrance. Therefore, we performed temperature-dependent gated four-point probe measurements from 10 to 300 K before and after both direct doping and remote doping (see figs. S13 and S14 for representative temperature-dependent conductance plots). Consequently, we extracted the temperature-dependent field-effect mobilities from the directly doped and remotely doped devices and decomposed them to charged impurity scattering–limited mobility and phonon scattering–limited mobility using Matthiessen’s rule. Figure 4 shows temperature-dependent field-effect mobilities calculated from the slope of the conductance plots at *V*_GS_ = 80 V for pristine MoS_2_ FETs (blue lines) and directly doped and remotely doped MoS_2_ FETs (red lines) with a different h-BN interlayer thickness (Fig. 4A for direct doping, Fig. 4B for remote doping with the 1 nm h-BN interlayer, and Fig. 4C for remote doping with the 2-nm h-BN interlayer). It should be noted that the mobility values after doping for all three devices shown in Fig. 4 were extracted under similar Δ*n* conditions (Δ*n* = 0.43, 0.45, and 0.38 × 10^12^ cm^−2^, respectively). The field-effect mobility from four-point probe measurements can be calculated using the following formula

$$\mu_{4\text{pp}}=(\mathrm{dG}/dV_{\text{GS}})\times (L/WC_{i})$$ (1)

where *G*, *L*, and *W* denote the conductance, channel length, and channel width, respectively. As shown in Fig. 4, the temperature-dependent mobilities of pristine MoS_2_ FETs, and directly and remotely doped MoS_2_ FETs increased as the temperature decreased because of suppressed phonon scattering (*51*). According to Matthiessen’s rule, the mobility of the channel can be written as

$$\mu_{4\text{pp}}(T)={\left(\frac{1}{\mu_{C}\;T^{\alpha }}+\frac{1}{\mu_{\text{ph}}T^{\beta }}\right)}^{-1}$$ (2)

where *T*, μ_c_, μ_ph_, and α and β denote temperature, charged impurity scattering–limited mobility, and phonon-limited mobility at the zero-temperature limit, and their exponents, respectively (*40*, *48*, *52*, *53*). In this analysis, we assumed that the scattering sources, excluding charged impurity and phonon, such as intrinsic defects and the roughness of the substrate, were negligible because the charged impurity and phonon scattering are the most dominant mechanisms in the charge transport of MoS_2_, as demonstrated previously (*48*, *51*). In terms of phonon scattering, the β value of the MoS_2_ device barely changed upon direct and remote doping compared with those of pristine devices, which suggests that the phonon scattering could be insensitive to the presence of the adsorbed dopants on the MoS_2_ surface. In addition, the extracted β value of the remotely doped MoS_2_ (β = −1.75) was slightly lower than that of the directly doped MoS_2_ (β = −1.95; see fig. S15 for more details), indicating that the top h-BN encapsulation can suppress the homopolar phonon scattering because the homopolar phonon mode is considered the most dominant among all phonon modes that contribute to phonon scattering in MoS_2_, as reported previously (*54*). The change in the charged impurity scattering–limited mobility (μ_c_*T*^α^) in mobility after doping can be interpreted by considering two major competing effects: the charge screening effect that enhances the mobility because of a larger screening of Coulomb potential created by the charged impurities (*55*) and larger charged impurity scattering because of a higher dopant ion density as a result of doping, which lowers the mobility because of the numerous charged impurities generated (*40*). In this particular range of doping, the increased mobility after doping indicates a greater screening effect than the scattering effect. Furthermore, previous studies have also shown similar mobility enhancement upon doping in MoS_2_ with BV molecules, which supports the observed mobility increase in our directly doped device (*47*–*51*, *55*–*57*).

In comparison, note that the μ_**c**_*T*^α^ values of remotely doped MoS_2_ devices (Fig. 4, B and C) are greater than that of the directly doped MoS_2_ device (Fig. 4A), indicating that the remotely doped devices have a lower degree of charged impurity scattering. In particular, μ_**c**_*T*^α^ of remotely doped MoS_2_ devices with the 2-nm h-BN interlayer was found to be even larger than that of the 1-nm h-BN case. This could be associated with the spatial separation between the additionally introduced charged impurities (in the form of the adsorbed dopants) and the MoS_2_ channel ensured by the thin h-BN interlayer in remote doping, which is expected to induce less Coulomb scattering by charged dopants. It is also meaningful to compare the mobility enhancement under different carrier concentration conditions (i.e., similar carrier concentrations in the pristine state, doped state, and similar Δ*n*). From our comparative analysis (see fig. S16 in section S16), we can deduce that the mobility enhancement of the remotely doped devices would be substantially larger than that of the directly doped devices in all cases. We also compared the mobility versus temperature behavior for directly and remotely doped devices when the screening effect from electrostatically accumulated carriers becomes minimal (i.e., the remaining carriers in the channel are mostly from BV doping; see fig. S17 in section S17 for more details). The relation between mobility and carrier density of MoS_2_ for direct and remote doping cases will be discussed in a later section.

### Enhanced mobility via suppressed charged impurity scattering in remote doping

The suppressed charged impurity scattering effect observed in the previously discussed remotely doped MoS_2_ FETs can be treated in a more quantitative manner. Ong and Fischetti (*58*) proposed a theoretical model for calculating the charged impurity–limited mobility (μ_imp_) of MoS_2_. In the model, the charged impurity scattering rate that is dependent on the charged impurity scattering potential (ϕ^scr^) determines the charged impurity–limited mobility of MoS_2_ FETs. While this model is successful in describing charged impurity scattering in a directly doped TMDC channel where the dopant ions are present on the surface of TMDC (*40*), the model has to be corrected for the remotely doped channel because of the spatial separation introduced by the h-BN interlayer. Considering the spatial separation, which places the charged impurities at a finite distance from the channel, the scattering potential in the model should be modified as the following equation (*59*)

$$\phi_{q}^{\text{remote}}=e^{-\mathrm{qd}}\phi_{q}^{\text{scr}}$$ (3)

where $\phi_{q}^{\text{remote}}$ and $\phi_{q}^{\text{scr}}$ are charged impurity scattering potential in the case of remote doping and direct doping, respectively, *d* is the h-BN thickness, and *q* is the scattering vector, defined as the magnitude of the difference in the scattered and initial wave vectors. A detailed discussion of the theoretical models is presented in fig. S18 and section S18.

**Fig. 5.**
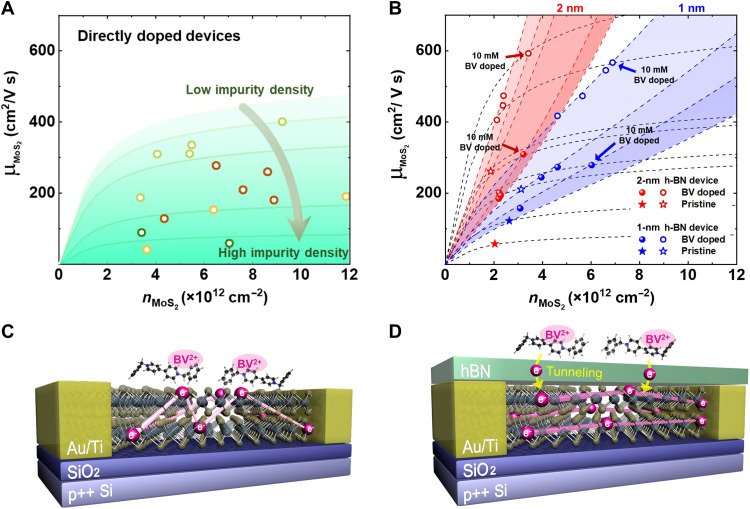
The suppressed charged impurity scattering effect in the remotely doped MoS_2_ FETs. Experimentally determined mobility (μ_MoS_2__) and calculated mobility (μ_imp_) as a function of carrier concentration (*n*_MoS_2__). The μ_imp_ values are calculated for (**A**) direct doping (green lines) and (**B**) remote doping (blue lines for the 1-nm h-BN interlayer and red lines for the 2-nm h-BN interlayer) for different impurity concentrations. μ_MoS_2__ and *n*_MoS_2__ values of remotely doped devices with the 1-nm (2 nm) h-BN interlayer are plotted as blue (red) circles and their pristine values are plotted as blue (red) stars, determined at 10 K to minimize the contribution of the phonon scattering. (**C**) The schematic images of the directly doped device that suffers from charged impurity scattering. (**D**) The schematic image of the remotely doped device in which the h-BN interlayer suppresses the charged impurity scattering induced by the remote BV^2+^ dopants.

To compare the experimentally obtained mobility values with the theoretical results, we calculated μ_imp_ as a function of carrier concentration for different impurity concentrations. The plotted lines in Fig. 5 (A and B) are the calculated μ_imp_ curves on the basis of the theoretical simulations in case of direct doping (green lines, Fig. 5A) and remote doping (red and blue lines, Fig. 5B) for different impurity concentrations, respectively. Regarding the remote doping, we plotted blue lines for *d* = 1 nm and red lines for *d* = 2 nm. For the experimental values, we used the mobility values at 10 K to minimize the contribution of the phonon scattering and compared only the charged impurity limited mobility. The mobility and carrier density values of directly doped devices are plotted as open circles and remotely doped devices with the 1-nm (2 nm) h-BN interlayer are plotted as blue (red) circles and their pristine values are plotted as blue (red) stars in Fig. 5 (A and B). According to our model, μ_imp_ increases with carrier density in the absence of the h-BN interlayer (i.e., direct doping), and the degree of increase in μ_imp_ decreases gradually (Fig. 5A). In contrast, the μ_imp_ plot for the h-BN case (i.e., remote doping) not only increases monotonically with carrier density but also shows a steeper increase when the spatial separation length, *d*, increases from 1 to 2 nm (see the blue and red lines for comparison in Fig. 5B, respectively). This shows that the larger spatial separation can further suppress charged impurity scattering, irrespective of the initial charged impurity density corresponding to the mobility values in pristine state. Therefore, unlike in the case of direct doping, the rate of increase in mobility is faster because of a dominant contribution from the charge screening effect as charge density increases.

Experimentally, the mobility values of directly doped MoS_2_ devices increased generally with the carrier density of MoS_2_, although the mobility values showed some fluctuations as the doping density increased (Fig. 5A). The general increase in the mobility observed in our MoS_2_ devices can be attributed to a larger contribution from the screening effect, which increases with doping strength. However, some fluctuations (see brown symbols in Fig. 5A, for example) were observed, which may be a manifestation of a complex interplay between the two competing effects (described above) that vary with doping: screening (i.e., increases mobility) and scattering (i.e., lowers mobility) effects. The observed fluctuations may result from a random nature of the spatial distribution of molecular dopants on the surface of the MoS_2_ channel, and they can be even intensified because of the formation of dopant clusters. Because each of these clusters can be treated as a multilayer stacking of the dopant molecules, the contribution of each dopant layer toward the overall amount of charge transfer (related to the charge screening effect) and degree of charged impurity scattering to the MoS_2_ channel would be different (*40*, *60*). To confirm such cluster formation of the BV dopants, we conducted AFM measurements. We could observe that BV dopants (formed as clusters) were distributed non-uniformly on the MoS_2_ surfaces, which supports the spatial inhomogeneity of the dopants (see fig. S19 for more details). Therefore, the observed fluctuations in the mobility could be due to complications induced by an unintentional spatial inhomogeneity of the dopants, which apparently limits the reliability of molecular SCTD via direct doping.

In contrast, the experimentally determined mobility of the remotely doped devices demonstrates a steep increase with the carrier density (Fig. 5B), which agrees with our theoretical predictions based on our modified charged impurity scattering model that incorporates the h-BN interlayer (red and blue lines). The steeper slope of the mobility with carrier density in the remotely doped devices relative to the directly doped devices represents a substantially reduced contribution from charged impurity scattering, as the spatial separation of the dopants from the MoS_2_ channel reduces the Coulomb potential exerted on the conducting electrons (see Eq. 3).

Although the model developed by Ong and Fischetti (*58*) cannot account for the physical separation of the dopants from the channel in the case of remote doping, it allows us to estimate the effective-charged impurity density (*n*_eff_) values for a quantitative comparison of remotely doped devices and directly doped devices. Because of a finite device-to-device variation, it is sensible to compare the change in the dopant-induced charged impurity density by comparing remotely doped devices with similar initial charged impurity density [i.e., a device with *n*_eff_ = 0.81 × 10^12^ cm^−2^ (4.00 × 10^12^ cm^−2^) indicated as a red open star (red filled star) versus a device with *n*_eff_ = 1.21 × 10^12^ cm^−2^ (2.00 × 10^12^ cm^−2^) indicated as a blue open star (blue filled star) in Fig. 5B]. The effective charged impurity densities of remotely doped devices (estimated from the fits shown as dashed lines in Fig. 5B) show smaller values (0.54 × 10^12^ cm^−2^ for 1-nm h-BN and 0.44 × 10^12^ cm^−2^ for 2-nm h-BN after doping with 10 mM) than those of directly doped devices (1.76 × 10^12^ cm^−2^ after doping with 10 mM), indicating that the magnitude of charged impurity scattering of remotely doped devices is lower than that of directly doped devices. This suppressed charged impurity scattering can partially account for markedly reduced fluctuations in the mobility curves observed for remote doping; the overall charged impurity scattering is reduced, and thus the varying degrees of charged impurity scattering from the random spatial distribution do not induce noticeable changes in the trend. This also indicates that the remote doping method can be a more reliable method for controlling the electrical properties of MoS_2_ devices than the direct doping method, even with solution-based doping methods that suffer from its intrinsically random nature to some extent.

Our discussion can be summarized by the schematic diagrams shown in Fig. 5 (C and D). Unlike the directly doped device, where electron conduction is hampered by the presence of charged BV dopants (BV^2+^) adsorbed directly on the surface of MoS_2_ and acting as charged impurities (Fig. 5C), the presence of the h-BN interlayer suppresses charged impurity scattering from the remote BV^2+^ dopants, thereby demonstrating the noninvasive manner of the remote doping concept presented in our study.

## DISCUSSION

In summary, we quantitatively investigated the remote charge transfer doping strategy for demonstrating the suppression of charged impurity scattering in MoS_2_ FETs. We realized remote charge transfer in MoS_2_ by simply inserting a thin h-BN layer between the MoS_2_ channel and molecular dopants, which was sufficient for achieving a notable charge transfer. With this remote charge transfer system, we systematically studied the suppression of the charged impurity scattering via performing temperature-dependent gated four-point probe measurements. As a result, we achieved substantially higher channel mobility, as well as more reliable doping controllability relative to the conventional direct doping method, which is supported by theoretical predictions. We believe that our study will pave the way for fully realizing the potential of remote doping to achieve a wide doping range without compromising the carrier mobility, which is required for high-performance emerging electronic and optoelectronic devices based on 2D vdW materials.

## MATERIALS AND METHODS

### Device fabrication

MoS_2_ and h-BN flakes were mechanically exfoliated from bulk MoS_2_ and h-BN crystals and transferred to a 270-nm SiO_2_/p++ Si substrate. Suitable MoS_2_ and h-BN flakes were located by using an optical microscope, and the thickness of the flakes were measured by an AFM system (NX 10, Park Systems).

After double electron resist layers [methyl methacrylate and poly(methyl methacrylate)] were spin-coated on the MoS_2_, the source-drain electrodes were patterned by using an electron beam lithography system (JSM-6510, JEOL). Subsequently, Ti (5 nm)/Au (45 nm) layers were deposited by using an electron-beam evaporator (KVE-2004 L, Korea Vacuum Tech).

To fabricate h-BN/MoS_2_ vdW heterostructures, we used the dry-transfer method as follows (*61*). The thin h-BN flakes were picked up by adhesive polycarbonate (purchased from Sigma-Aldrich, PC) deposited on the dome-shaped polydimethylsiloxane stamp. Then, the h-BN flake was placed on top of the fabricated MoS_2_ FETs. The heterostructure was dipped into chloroform overnight to remove the remaining polymer residue. Last, we annealed the device at 200°C in an argon atmosphere for 2 hours to improve the interfaces before performing the electrical measurement.

### Electrical characterization and temperature-dependent measurements

To confirm SCTD in the MoS_2_ devices, two-point probe electrical characterizations were performed in a temperature-variable probe station (MSTECH, M6VC) using a semiconductor parameter analyzer (Keithley 4200-SCS). Keithley 4200-SCS with a pre-amplifier was used to measure the voltage drop across the channel by applying constant dc bias for four-point probe measurements. The temperature-dependent measurements of the directly and remotely doped FETs were carried out with a cryostat system (CS204*I-FMX-12, Advanced Research Systems).

### SCTD treatment

We used a drop-casting method with BV solution to perform surface molecular charge transfer doping. For the preparation of the BV solutions, we referred to the previous study (*62*). The BV dichloride (16.35 mg; purchased from Sigma-Aldrich) was dissolved in deionized water (4 ml) and then we added toluene (4 ml; purchased from Sigma-Aldrich). Sequentially, sodium borohydride (40 mg; purchased from Sigma-Aldrich) as a reducing agent was added to the deionized water/toluene layered solution, which was then stirred overnight. While the chemical reaction proceeded, a color change from purple to yellow was observed in the toluene layer. When the chemical reaction was almost completed and the color of the toluene solution was stabilized in yellow, the upper toluene layer was extracted with a micropipette. To conduct the experiments in various doping ranges, we diluted 10 mM BV solution into 1, 2.5, and 5 mM with toluene, respectively. In the drop-casting process, we used 20 μl of the BV solution and we waited for 10 min for the solvent to evaporate under ambient conditions.

## Supplementary Materials

### This PDF file includes:

Sections S1 to S19 Figs. S1 to S19 Tables S1 and S2 References
