# Supplementary material for: Reduced dopant-induced scattering in remote charge-transfer-doped MoS2 field-effect transistors
Source: Sci Adv. 2022 Sep 21;8(38):eabn3181. doi: 10.1126/sciadv.abn3181 (PMC9491718; doi:10.1126/sciadv.abn3181)
Supplement: Supplementary file 1 — Sections S1 to S19 Figs. S1 to S19 Tables S1 and S2 References [file sciadv.abn3181_sm.pdf]

Science Advances
AAAS

Supplementary Materials for

Reduced dopant-induced scattering in remote charge-transfer-doped MoS_2 field-effect transistors

Juntae Jang *et al.*

Corresponding author: Keehoon Kang, keehoon.kang@snu.ac.kr; Kyungjune Cho, kcho@kist.re.kr; Takhee Lee, tlee@snu.ac.kr

Sci. Adv. **8**, eabn3181 (2022)
DOI: 10.1126/sciadv.abn3181

This PDF file includes:

Sections S1 to S19
Figs. S1 to S19
Tables S1 and S2
References

Supplementary Materials for

Reduced dopant-induced-scattering in remote charge transfer doped MoS_2 field-effect transistors

Juntae Jang *et al.*

Corresponding author: Takhee Lee, tlee@snu.ac.kr

Table of Contents

Section S1. Device fabrication of remotely doped MoS_2 FETs

Section S2. Atomic force micrograph of MoS_2 and h-BN flakes used for devices

Section S3. Scanning tunneling electron micrograph of h-BN/MoS_2 heterostructures

Section S4. Encapsulation of thin h-BN on MoS_2 FETs

Section S5. Thickness determination of thick h-BN flakes

Section S6. h-BN interlayer thickness dependence on remote charge transfer doping

Section S7. Ambient stability of remote charge transfer doping

Section S8. Contact resistances of directly doped and remotely doped devices

Section S9. Offset-correction process

Section S10. Doping controllability of BV-doped FETs characterized by two-point probe measurement

Section S11. Normalized σ_{4pp} values of directly doped and remotely doped devices

Section S12. The estimated final carrier density after doping for directly and remotely doped devices

Section S13. Temperature-dependent conductance of MoS_2 FETs without h-BN

Section S14. Temperature-dependent conductance of h-BN/MoS_2 FETs

Section S15. Components of Matthiessen's rule for directly doped and remotely doped devices

Section S16. Comparison of directly and remotely doped devices under similar carrier concentration conditions

Section S17. Comparison of directly and remotely doped devices under minimal screening from the gate-induced carriers

Section S18. Theoretical models for calculating the mobility of directly doped and remotely

doped MoS_2

Section S19. Spatial inhomogeneity of the BV dopants on the MoS_2 surfaces

Figures S1-S19

Tables S1-S2

Section S1. Device fabrication of remotely doped MoS_2 FETs

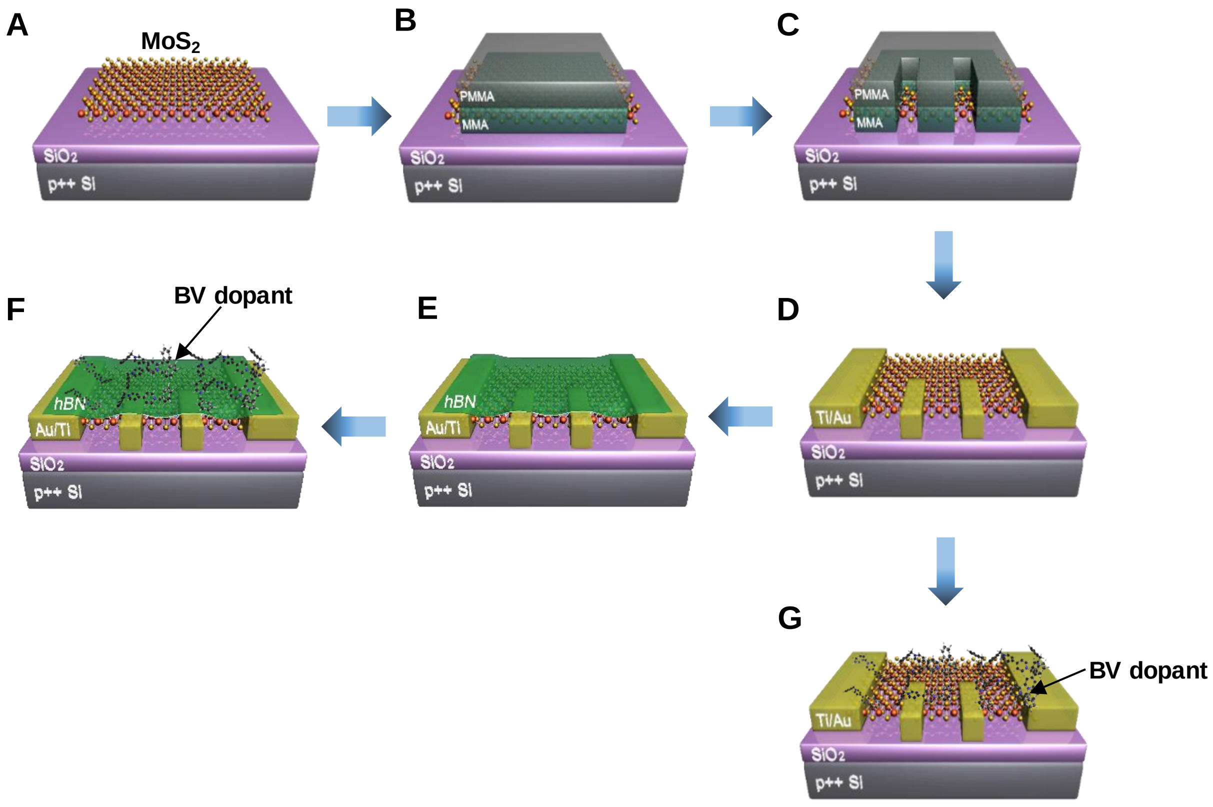

Fig. S1. Fabrication processes for the MoS_2 FETs and h-BN/MoS_2 heterostructure FETs. (**A**) MoS_2 flakes of thickness less than ~5 nm are prepared by the mechanical exfoliation method. (**B**) Electron resist double layers of methyl methacrylate (MMA, 9 wt% concentration in ethyl lactate) and polymethyl methacrylate (PMMA, 950,000 molecular weight, 5 wt% concentration in anisole) are spin-coated at 4000 rpm for 50 s, each followed by hard baking at 180 °C for 90 s. (**C**) Electron-beam lithography is performed for patterning four-point probe electrodes. (**D**) Metal electrodes of Au (50 nm)/Ti (5 nm) are deposited by an electron-beam evaporator. (**E**) After the metal deposition process, a thin h-BN layer is transferred by the dry transfer method using dome-shaped polydimethylsiloxane (PDMS) stamp coated by a polycarbonate (PC) film. (**F**) Benzyl viologen (BV) molecules are deposited onto the h-BN

layer for remote charge transfer doping (**G**) Directly onto MoS_2 FETs (without h-BN layer) for direct doping.

Section S2. Atomic force micrograph of MoS_2 and h-BN flakes used for devices

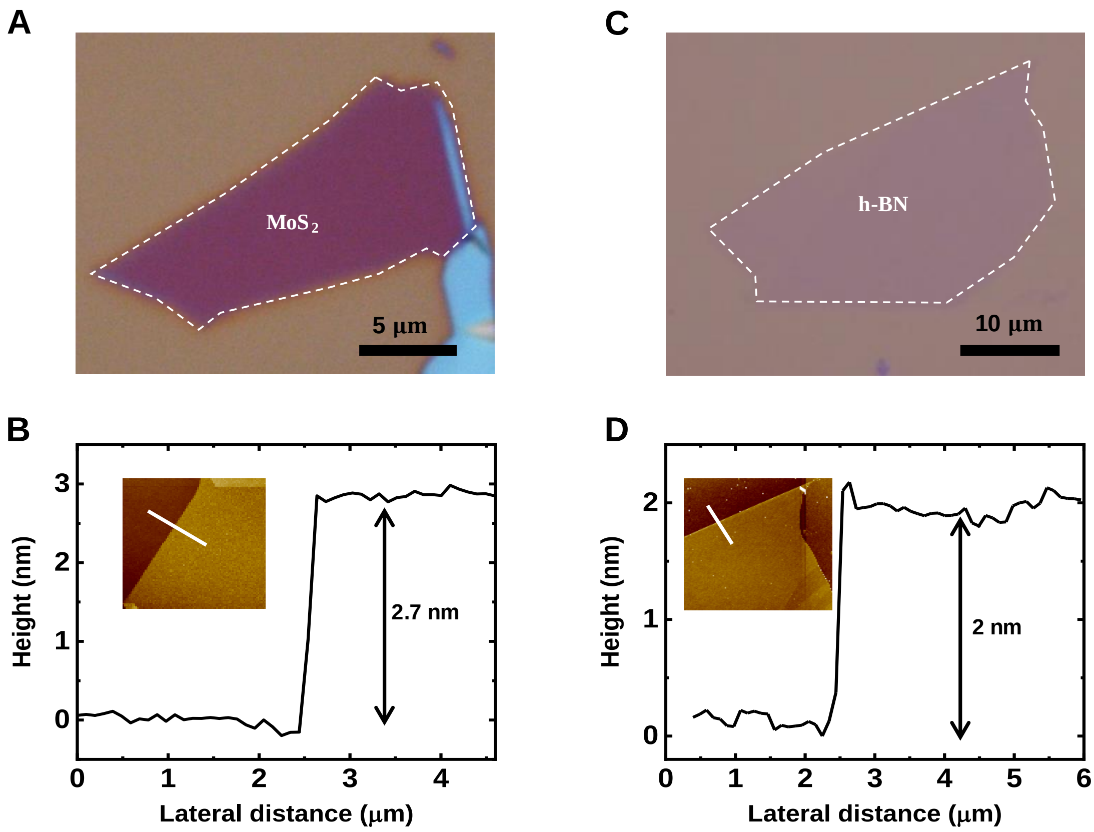

Fig. S2. The optical images of MoS_2 and h-BN flakes and atomic force microscopy (AFM) height profiles for determining the thickness of flakes. (**A**) MoS_2 flake which was used in Fig. 1B of the main manuscript. (**B**) The thickness of the MoS_2 flake is ~2.7 nm (four layers) measured by AFM (NX 10 AFM, Park Systems) (**C**) The optical image of a thin h-BN flake for remote doping. Especially, we selected h-BN flakes of thickness less than ~3 nm to achieve high doping efficiency. (**D**) The thickness of this h-BN layer was found to be ~2 nm.

Section S3. Scanning tunneling electron micrograph of h-BN/MoS_2 heterostructures

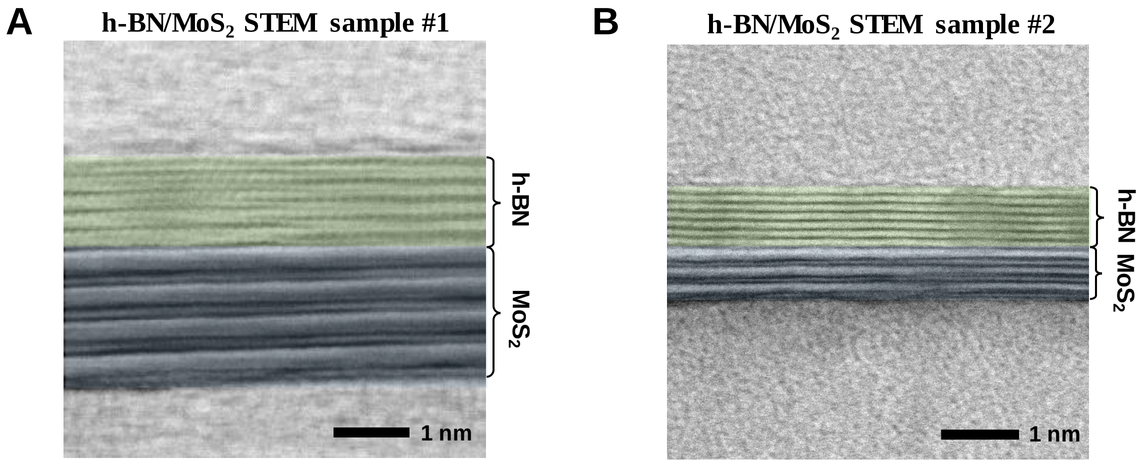

Fig. S3. Spherical aberration-corrected scanning transmission electron microscopy images of thin h-BN/MoS_2 samples. (**A**) Device sample #1 consists of five-layer h-BN and four-layer MoS_2 and device sample (**B**) Device sample #2 consists of seven-layer h-BN and trilayer MoS_2. From these images, we could assure that our fabricated devices have well-stacked heterostructures. We used the dry-transfer method to fabricate thin h-BN/MoS_2 samples.

Section S4. Encapsulation of thin h-BN on MoS_2 FETs

To confirm that a thin h-BN layer can encapsulate MoS_2 FETs properly, we investigated the electrical hysteresis of MoS_2 FETs without the h-BN layer (black line) and covered with ~ 2 nm thin h-BN layer (blue line) under ambient conditions, as shown in Fig. S4. Since one of the dominant origins of the hysteresis is the adsorption of water and oxygen molecules from the ambient environment (*11, 63, 64*). we can observe a remarkably smaller hysteresis window in the MoS_2 FET encapsulated with the h-BN layer compared to un-encapsulated MoS_2 FETs. Thus, we confirmed that ~2 nm-thick h-BN layer can provide a sufficient encapsulation effect, separating the dopant molecules from the channel successfully. However, it should be noted that during the dry-transfer process, air can be trapped between the h-BN interlayer and MoS_2 channel. Even if such blisters are removed as much as possible by vacuum thermal annealing, they may not be removed completely. Thus, we presume the blisters can act as the charged impurity scattering sources, which results in a smaller current value in the encapsulated MoS_2 FETs compared to that of the device before encapsulation in Fig. S4.

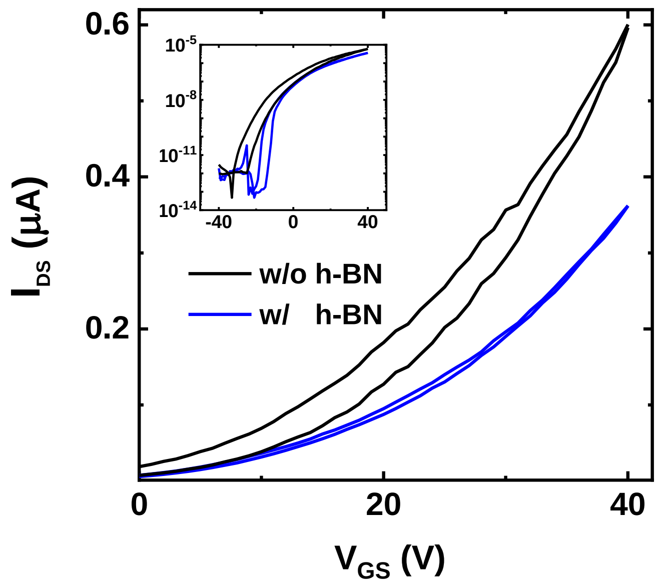

Fig. S4. Electrical hysteresis of the MoS_2 FETs with and without h-BN layer. Black solid line indicates the electrical hysteresis of MoS_2 FETs without the h-BN layer. Blue solid line shows the electrical hysteresis of MoS_2 FETs covered with ~ 2 nm thin h-BN layer. Both devices were measured under ambient condition.

Section S5. Thickness determination of thick h-BN flakes

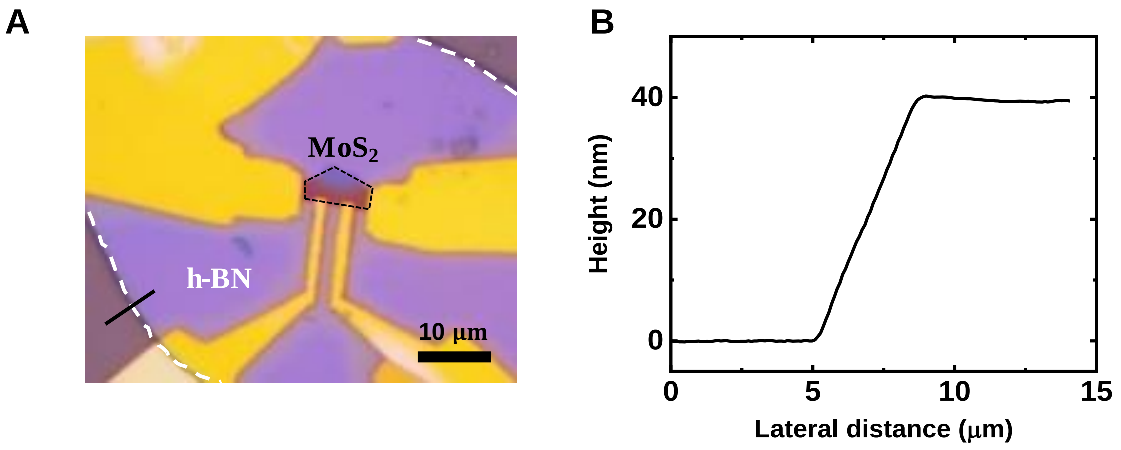

Fig. S5. Thickness determination of thick h-BN flakes. We also fabricated MoS_2 FETs covered with a thick h-BN to confirm extremely low doping efficiency with a high spatial distance between the dopant molecules and the channel. (**A**) The optical image of a thick h-BN/MoS_2 FET which was used in Fig. 2E of the main manuscript. (**B**) The thickness of the thick h-BN flake is ~ 40 nm. The thickness of the h-BN flakes was measured by AFM.

Section S6. h-BN interlayer thickness dependence on remote charge transfer doping

To investigate the critical thickness of h-BN, we fabricated additional devices with varying the thickness of h-BN and characterized them. Figures S6A and B below show the increased carrier density (Δn) *versus* the thickness of the h-BN interlayer with a linear scale and logarithmic scale of Δn, respectively. The exponential decay of Δn with increasing thickness of the h-BN interlayer is expected from the decreasing charge tunneling probability through the h-BN interlayer.

As shown in Figs. S6A and B, the values of Δn are explained by exponential fitting curves (dashed lines) as a form of exp(-t/τ), where t is the thickness of the h-BN interlayer and τ is a characteristic length for the remote charge transfer doping which is extracted to be ~3.7 nm. This value is reasonable considering tunneling-based charge transfer effect (*65*).

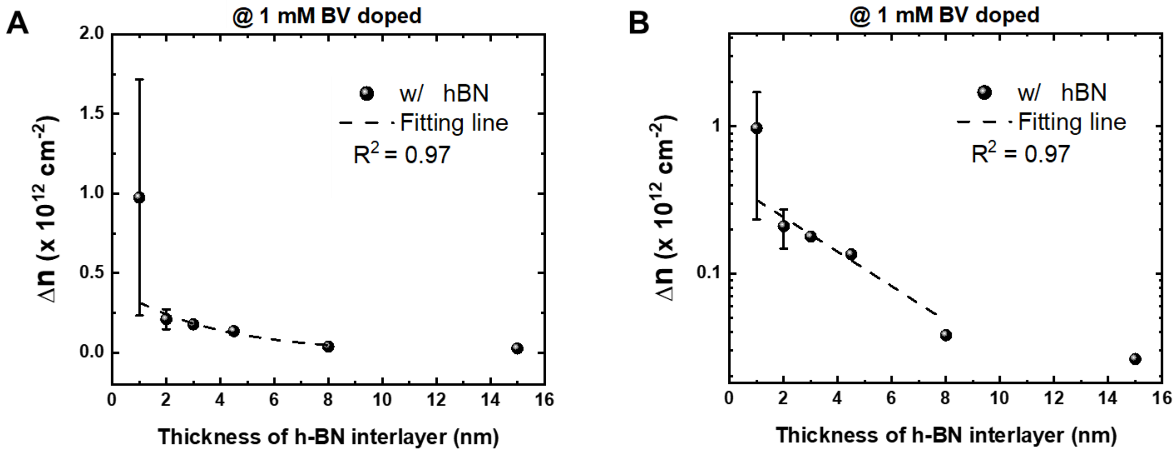

Fig. S6. The increased carrier density (Δn) *versus* the thickness of the h-BN interlayer. (A) a linear scale and **(B)** a logarithmic scale. The data point of 15 nm-thick h-BN interlayer is not included in the fitting because Δn is negligible in the error range.

Section S7. Ambient stability of remote charge transfer doping

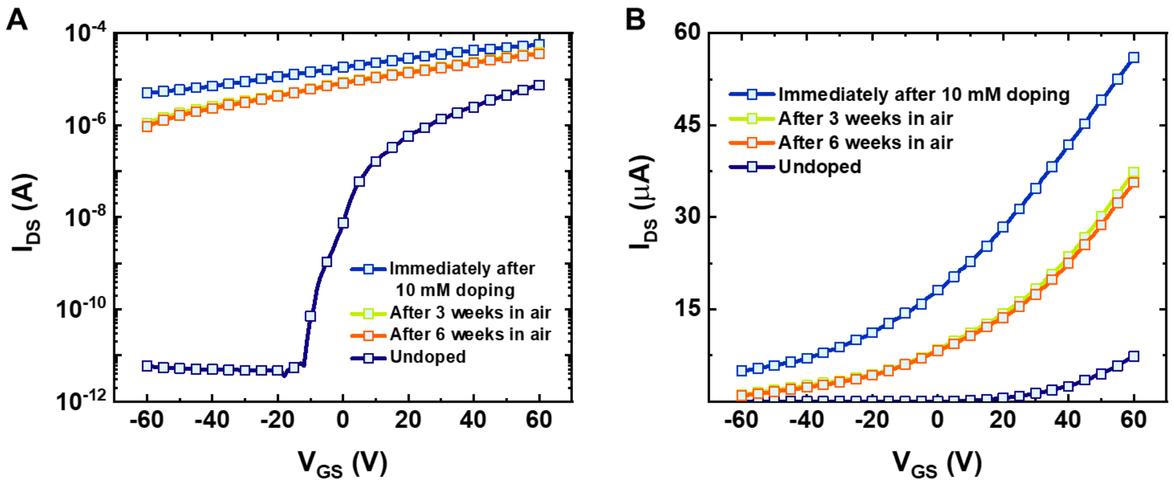

Fig. S7. The I_{DS} of pristine and remotely doped devices in time (3 and 6 weeks later after doping). (A) a logarithmic scale and **(B)** linear scale. We characterized the transfer curves of the remotely doped devices before and after doping at 300 K in a vacuum. Even though the I_{DS} level of devices after 3 and 6 weeks somewhat decreased compared to the initial condition (i.e., the current level of the devices immediately after the 10 mM doping), the remote doping effect was still identified even after 3 and 6 weeks.

Section S8. Contact resistances of directly doped and remotely doped devices

For obtaining contact resistance (R_c) of directly and remotely doped devices before and after BV doping, we conducted two-point and four-point probe measurements. Fig. S8 shows R_c as a function of V_{GS}, extracted from the equation $R_c = \frac{1}{2}\left(R_{2pp} - R_{4pp}\frac{l_{tot}}{l_{in}}\right)$, where R_{2pp} and R_{4pp} are the two-point and four-point probe resistance, respectively, and l_{tot} and l_{in} are the total and inner channel lengths, respectively. In Fig. S8, R_c values were extracted from pristine (undoped) devices and doped devices with 5 mM BV solution. As shown in Fig. S8, the R_c values of the directly and remotely doped devices were found to be much smaller than those of the pristine devices. The slight discrepancy between the trends shown in Fig. 3A and Fig. S10A could arise from the varied contact resistance due to the BV doping.

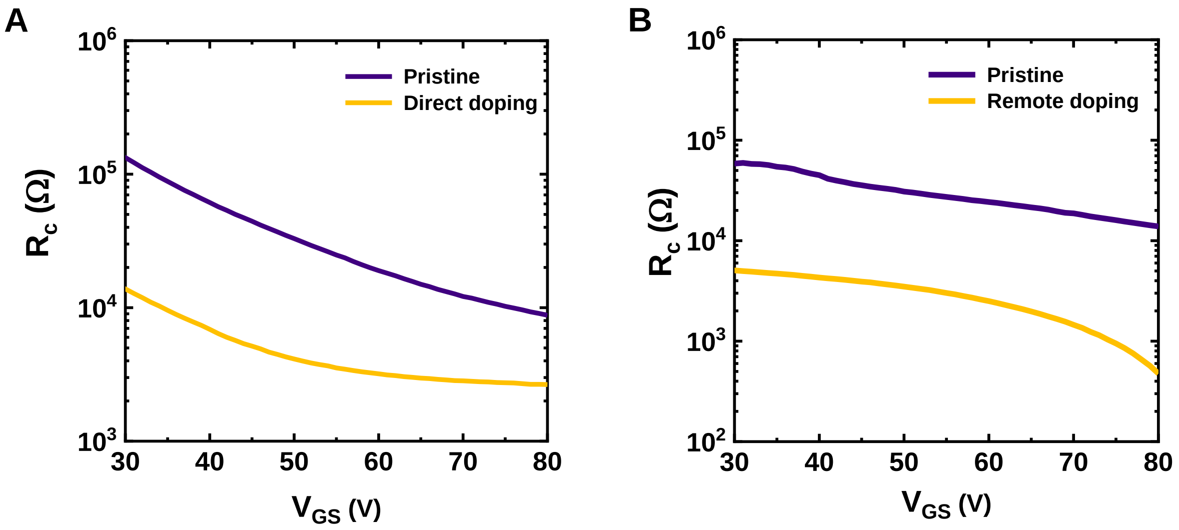

Fig. S8. Contact resistances (R_c) as a function of V_{GS}. (A) directly and **(B)** remotely doped devices before and after BV doping. The R_c values of the directly and remotely doped devices were found to be much smaller than those of the pristine devices. The R_c values were extracted from pristine (undoped) devices and doped devices with 5 mM concentration of BV solution.

Section S9. Offset-correction process

We performed offset-correction analysis for the data acquired by four-point probe measurements due to the limited voltage resolution of the measurement equipment. Fig. S9A shows the four-point probe conductance (σ_{4pp}) of remotely doped MoS_2 FETs as a function of V_{GS} before offset-correction with varying concentrations of BV solutions. As shown in this figure, there are unclear peaks in the conductance curve at the off-state, i.e., at V_{GS} from 20 V to 50 V. Since the semiconductor parameter analyzer (Keithley 4200-SCS) used in this study has a 0.2 μV resolution and the resistance of the channel at the off-state is significantly high, the voltage probe does not measure the voltage difference values between V_1 and V_2 electrodes properly at the off-state. Thus, we did an offset-correction to address such an issue as described below.

Fig. S9A shows the σ_{4pp} values in a remotely doped device before offset-correction as doping concentration increases from 1 to 10 mM. In Fig. S9B, the line with black square symbols indicates the voltage difference (ΔV) values between the inner voltage probes versus V_{GS} before offset-correction, and the line with red circle symbols indicates ΔV values after offset-correction. Before the offset correction, a significantly small value of $\Delta V \sim 0$ V entered the denominator of conductance $G = (dI/d\Delta V)$, resulting in a bizarre divergence peak in conductance. To address it, we subtracted a small value of 0.03 V to all ΔV, shifting it downward so that it would not pass through $\Delta V = 0$ V. Fig. S9C shows the σ_{4pp} values as doping concentration increases from 1 to 10 mM after the offset-correction for the same data shown in Fig. S9A. Note that the σ_{4pp} values were lowered by less than 3% after the offset-correction at $V_{GS} = 80$ V where we obtained the μ_{4pp} and this does not affect the scientific discussions of the experimental results.

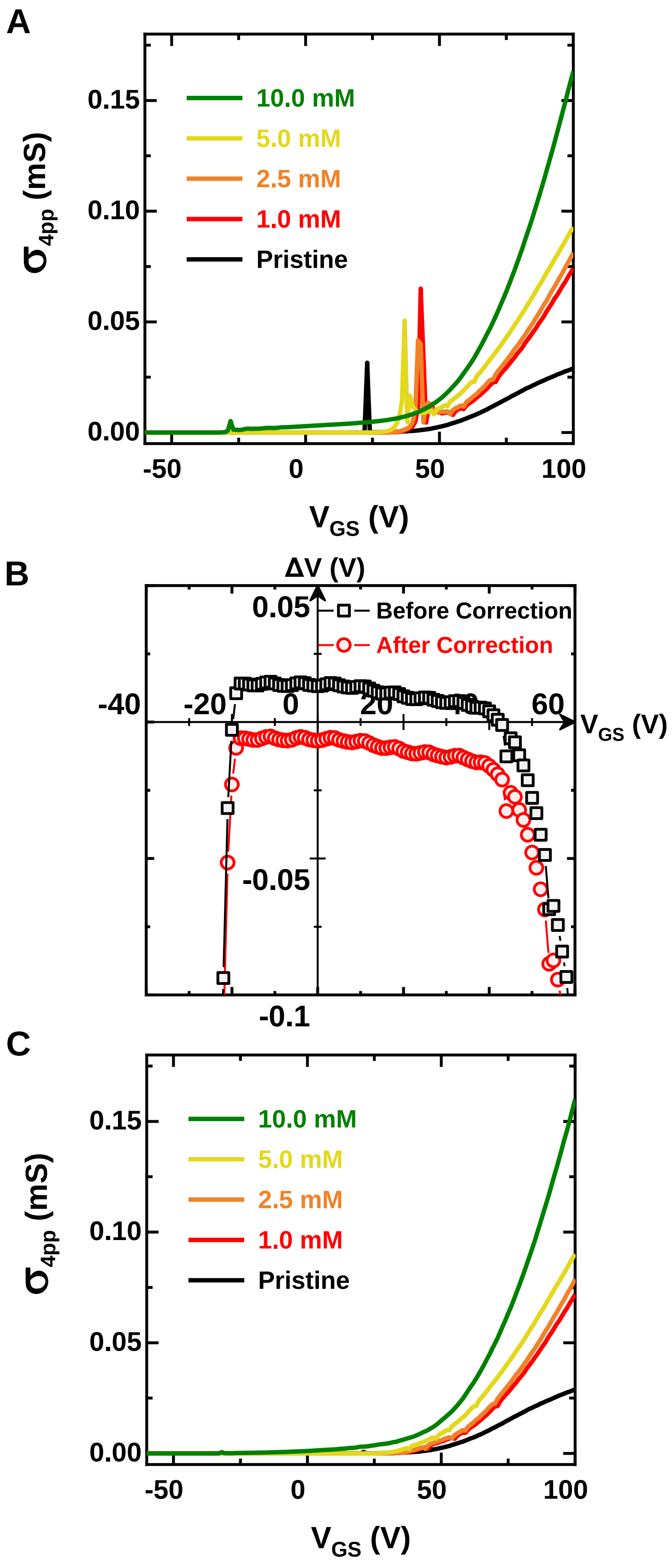

Fig. S9. Offset-correction process. The four-point probe conductance (σ_{4pp}) of remotely

doped MoS_2 FETs as a function of V_{GS} (**A**) before and (**C**) after offset-correction with varying concentrations of BV solutions. (**B**) The lines with black square and red circle symbols indicate the voltage difference (ΔV) values between the inner voltage probes versus V_{GS} before and after offset-correction, respectively. Kinks are observed (**A**) before the offset-corrections however, kinks disappear (**C**) after the offset-correction process. An excessive offset correction could result in inaccuracies towards the extraction of the mobility values and therefore we limited the offset correction to an extent such that the resulting σ_{4pp} values were lowered by less than 3% after the offset-correction at V_{GS} = 80 V where we obtained the $\mu_{4pp.}$

Section S10. Doping controllability of BV-doped FETs characterized by two-point probe measurement

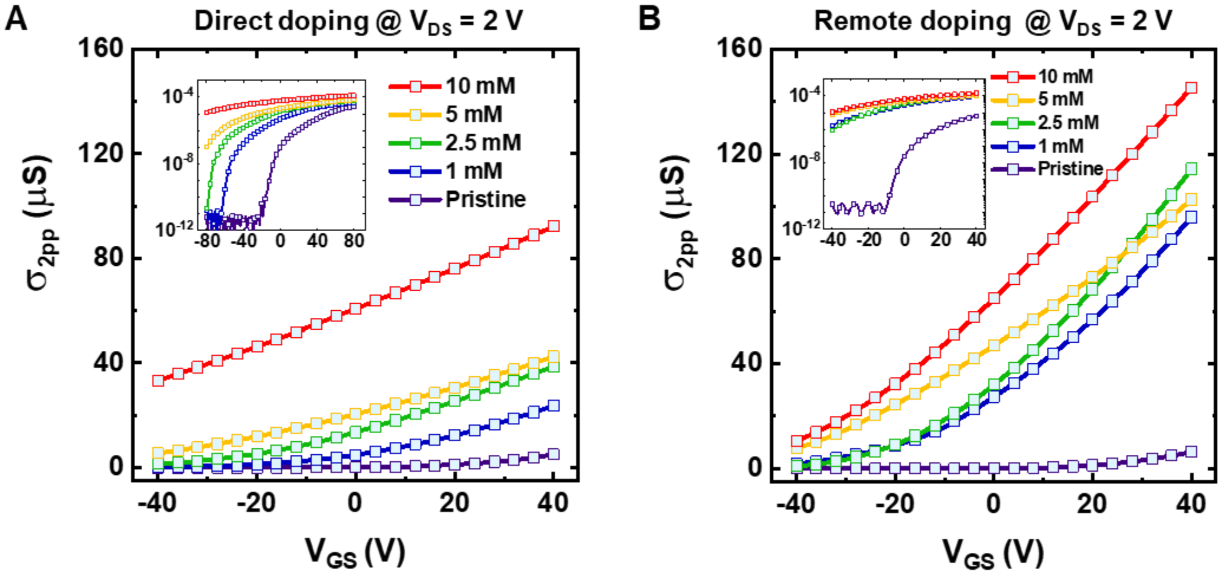

Fig. S10. Doping controllability of BV doped FETs characterized by two-point probe measurement. (**A**) The two-point probe conductance (σ_{2pp}) versus gate voltage (V_{GS}) of directly doped and (**B**) Remotely doped MoS_2 FETs with doping concentrations varying from 1 to 10 mM. In particular, we characterized the MoS_2 FETs at 10 K in a vacuum to avoid unwanted effects from the ambient and the phonon scattering. For both directly and remotely doped devices, the σ_{2pp} increased and the threshold voltages shifted to the negative gate voltage direction as the doping concentration increased, which indicates that the Fermi level of remotely and directly doped MoS_2 channels can be modulated.

Section S11. Normalized σ_{4pp} values of directly and remotely doped devices

Fig. S11 shows normalized σ_{4pp} versus doping concentration plots for directly and remotely doped devices. From Figs. 3A and 3B, we extracted the σ_{4pp} values for doped devices (σ_{doped}) and undoped devices ($\sigma_{pristine}$), respectively, at V_{GS} = 40 V for each doping concentration and calculated the normalized σ_{4pp} values which are defined as the ratio of σ_{doped} to $\sigma_{pristine}$. As shown in Fig. S11, we could quantitatively identify the increase in conductance of directly and remotely doped devices for each doping concentration through the normalized σ_{4pp}. In other words, the normalized σ_{4pp} values of the remotely doped devices (red circles) are significantly improved compared to those of the directly doped devices (blue circles) for each doping concentration. Consequently, this result clearly suggests the benefit of remote charge transfer doping by suppressing the charged impurity scattering caused by charge transfer doping.

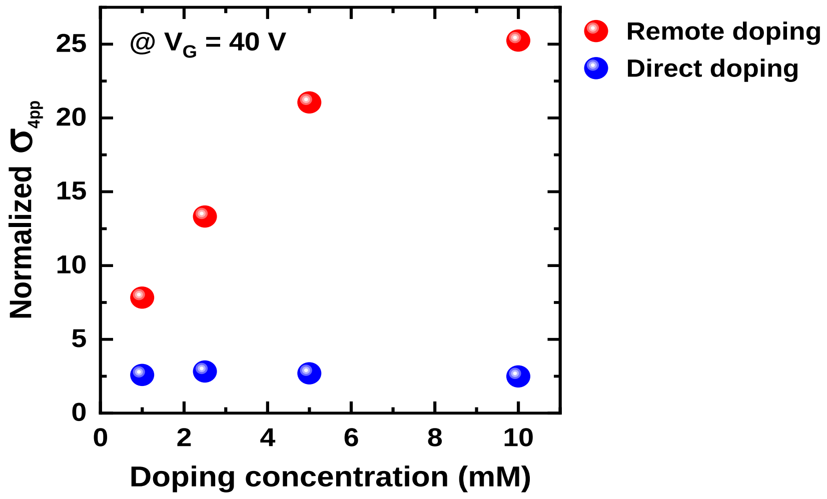

Fig. S11. Normalized σ_{4pp} values of directly and remotely doped devices. The normalized σ_{4pp} values of the remotely doped devices are significantly larger than those of the directly doped devices for each doping concentration.

Section S12. The estimated final carrier density after doping for directly and remotely doped devices

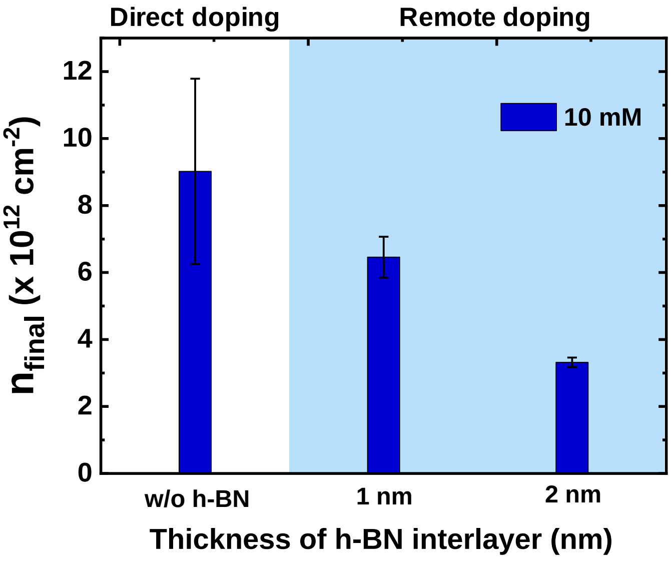

Fig. S12. The estimated n_{final} for directly and remotely doped FET devices with 10 mM concentrations of BV solution from the electrical data measured at 10 K. The left column indicates n_{final} in directly doped devices. The middle and the right column represent n_{final} in remotely doped devices with 1 nm and 2 nm h-BN remotely doped device, respectively. The error bars were determined statistically by characterizing several devices (7 devices with direct doping and 4 devices with remote doping). The average n_{final} value of the directly doped devices (9.02×10^{12} cm^{-2}) is higher than those of the remotely doped devices (1 nm h-BN: 6.46×10^{12} cm^{-2}, 2 nm h-BN: 3.32×10^{12} cm^{-2}). This means that the larger spatial separation by the h-BN interlayer leads to the smaller doping efficiency.

Section S13. Temperature-dependent conductance of MoS_2 FETs without h-BN

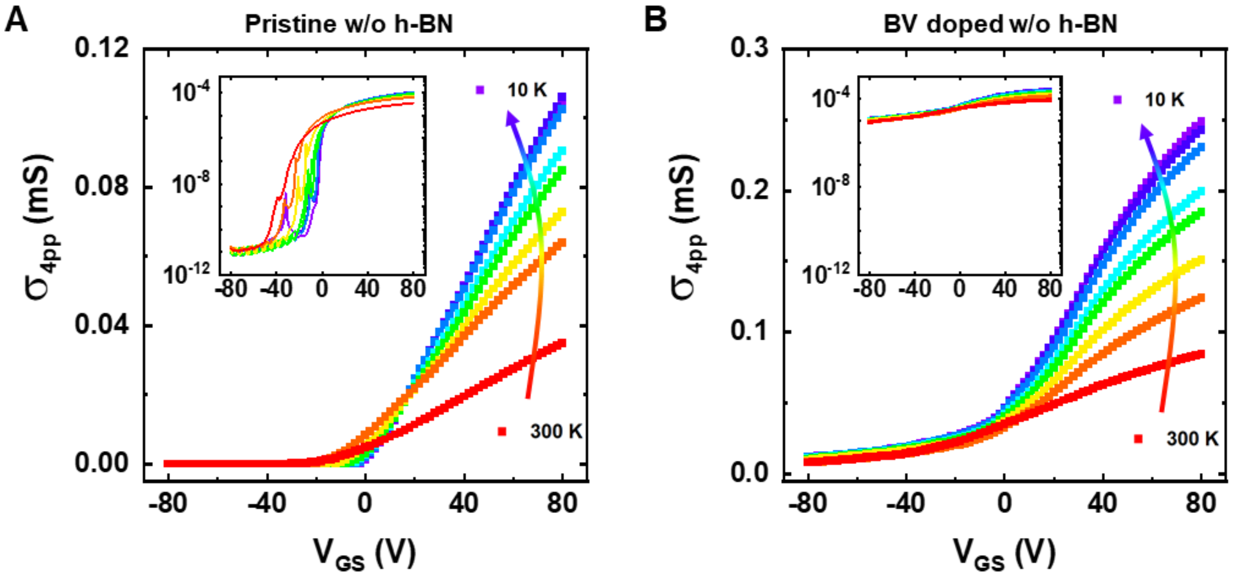

Fig. S13. Temperature-dependent conductance of MoS_2 FETs without h-BN. (**A**) The temperature-dependent σ_{4pp} values of MoS_2 FETs (without h-BN layer) as a function of V_{GS} before BV doped at the temperature range from 10 to 300 K (**B**) The temperature-dependent σ_{4pp} values of MoS_2 FETs (without h-BN layer) as a function of V_{GS} after BV doped with 1 mM BV solution at the temperature range from 10 to 300 K. As the temperature decreased, the threshold voltage shifted to the positive gate voltage direction, meaning that the carrier concentration decreased due to reduced thermal generation. Additionally, as the temperature decreased, the subthreshold swing values decreased as shown in Fig. S13A inset (see the slope increased as the decreasing temperature) and conductance levels were enhanced due to the suppressed phonon scattering (*40*).

Section S14. Temperature-dependent conductance of h-BN/MoS_2 FETs

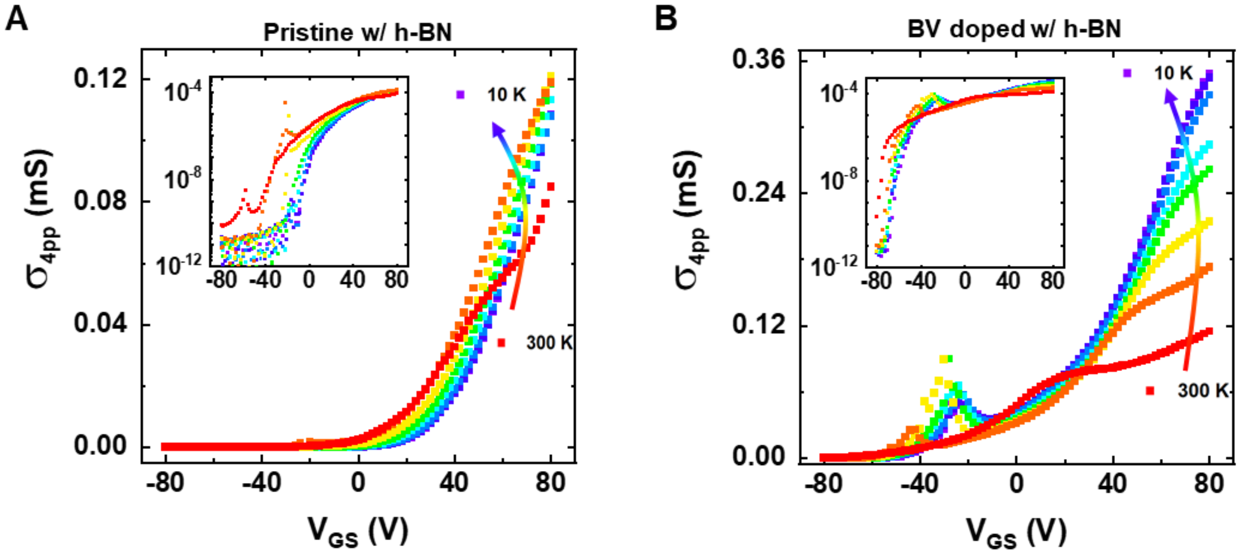

Fig. S14. Temperature-dependent conductance of h-BN/MoS_2 FETs. (**A**) The temperature-dependent σ_{4pp} values of h-BN/MoS_2 FETs before doping at the temperature range from 10 to 300 K. (**B**) The temperature-dependent σ_{4pp} values of remotely doped MoS_2 FETs as a function of V_{GS} with 1 mM BV solution at the temperature range from 10 to 300 K. Both devices show as the temperature decreased, the threshold voltages shifted to the positive gate voltage direction and the subthreshold swing values decreased (see the slope increased as the decreasing temperature) and the conductance levels were enhanced.

Section S15. Components of Matthiessen's rule for directly doped and remotely doped devices

Table S1 summarizes the components from the mobility values, decomposed by Matthiessen's rule (see Eq. (2) in the main manuscript), of directly and remotely doped MoS_2 FETs in Fig. S15. In Table S1, the β value was barely changed after BV treatment for both directly and remotely doped devices, which indicates that the homopolar phonon contribution to the charge transport of MoS_2 FETs was not significantly changed after BV doping (*54*). However, the μ_c increased for directly and remotely doped devices. The μ_c increment relative to its pristine of remote doping is higher than that in the case of direct doping, which means the thin h-BN layer suppresses the charged impurity scattering and it makes remotely doped devices has higher mobility than directly doped devices at 10 K.

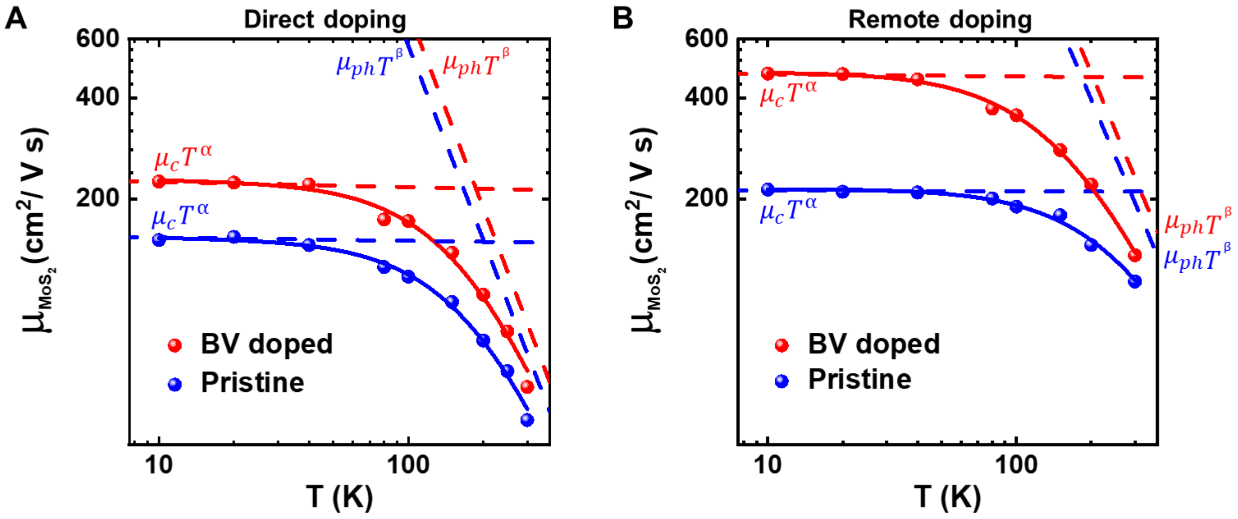

Fig. S15. Temperature-dependent mobility measurements. Temperature-dependent mobility values of (**A**) directly and (**B**) remotely doped devices at temperatures from 10 to 300 K. The mobility values were extracted from the four-point probe conductance plots at V_{GS} = 80 V for pristine devices (blue lines) and doped devices (red lines).

Table S1. Matthiessen's rule component values.

Direct doping	μ_C (cm^2/ V s)	μ_{ph} (cm^2/ V s)	α	β
**Pristine**	157.01	4.07×10^6	−0.001	−1.93
**Doped**	233.86	5.78×10^6	−0.011	−1.95

Remote doping	μ_C (cm^2/ V s)	μ_{ph} (cm^2/ V s)	α	β
**Pristine**	213	5.79×10^6	0.009	−1.73
**Doped**	479.37	4.22×10^6	0.015	−1.75

Section S16. Comparison of directly and remotely doped devices under similar carrier concentration conditions

To systematically investigate and compare the mobility enhancement after the doping under similar carrier concentration conditions, we present three cases: i) similar $n_{pristine}$, ii) similar n_{doped}, and iii) similar Δn, as follows.

Case 1. Similar $n_{pristine}$: Directly doped vs remotely doped devices with similar carrier concentrations in the *pristine* state.

- The carrier concentration of the pristine state ($n_{pristine}$) is 3.20 × 10^{12} cm^{-2} for the directly doped device (Fig. S16A) and 3.12 × 10^{12} cm^{-2} for the remotely doped device (Fig. S16C). In this pristine state, the charged impurity scattering-limited mobility ($\mu_c T^{\alpha}$) of the directly doped device (213.6 $cm^2/V{\cdot}s$) is greater than that of the remotely doped device (212.2 $cm^2/V{\cdot}s$). After that, we doped both devices with 1 mM BV concentration and the carrier concentration of the doped state (n_{doped}) is found to be 6.39 × 10^{12} cm^{-2} for the directly doped device and 4.62 × 10^{12} cm^{-2} for the remotely doped device. However, we note that the $\mu_c T^{\alpha}$ of the remotely doped device (472.0 $cm^2/V{\cdot}s$) is greater than that of the directly doped device (244.7 $cm^2/V{\cdot}s$).

Case 2. Similar n_{doped}: Directly doped vs remotely doped devices with similar carrier concentrations in the *doped* state.

- The $n_{pristine}$ is 4.47 × 10^{12} cm^{-2} for the directly doped device (Fig. S16B) and 3.12 × 10^{12} cm^{-2} for the remotely doped device (Fig. S16C). In this pristine state, the $\mu_c T^{\alpha}$ of the directly doped device (315.3 $cm^2/V{\cdot}s$) is greater than that of the remotely doped device (212.2 $cm^2/V{\cdot}s$). Likewise, we doped both devices with 1 mM and then, n_{doped} is found to be 4.90

× 10^{12} cm^{-2} for the directly doped device and 4.62 × 10^{12} cm^{-2} for the remotely doped device. Similar with the previous result, the $\mu_c T^{\alpha}$ of the remotely doped device (472.0 $cm^2/V{\cdot}s$) is greater than that of the directly doped device (331.3 $cm^2/V{\cdot}s$).

Case 3. Similar Δn: Directly doped vs remotely doped devices with *similar change* in the *carrier concentrations* via doping.

- We compared the directly and remotely doped devices for Δn = 0.43 × 10^{12} cm^{-2} (Fig. S16B) and Δn = 0.38 × 10^{12} cm^{-2} (Fig. S16D), respectively. In this pristine state, the $\mu_c T^{\alpha}$ of the directly doped devices (315.3 $cm^2/V{\cdot}s$) is larger than that of the remotely doped devices (230.7 $cm^2/V{\cdot}s$). Likewise, in doped state, the $\mu_c T^{\alpha}$ of the remotely doped devices (480.5 $cm^2/V{\cdot}s$) is greater than that of the directly doped devices (331.3 $cm^2/V{\cdot}s$). This corresponds to ~108% enhancement in average in the mobility value for the 2 nm remotely doped device, which is 21 times increase in the mobility enhancement than the directly doped device (~5% enhancement in average). Therefore, we can deduce that the mobility enhancement of the 2 nm remotely doped devices is significantly larger than that of the directly doped devices under similar Δn conditions.

The following table summarizes the above comparisons between directly doped and remotely doped devices.

Table S2. **The mobility enhancement values (i.e. the change in mobility by doping/initial mobility) for each case of similar charge concentration conditions, for directly and remotely doped devices.**

	Mobility enhancement (%)		
	Direct doping	1 nm h-BN remote doping	2 nm h-BN remote doping
**Case 1: Similar $n_{pristine}$**	14 % ($n_{pristine}$ = 3.20 × 10^{12} cm^{-2})	122 % ($n_{pristine}$ = 3.12 × 10^{12} cm^{-2})	-
**Case 2: Similar n_{doped}**	5 % (n_{doped} = 4.90 × 10^{12} cm^{-2})	122 % (n_{doped} = 4.62 × 10^{12} cm^{-2})	-
**Case 3: Similar Δn**	5 % (Δn = 0.43 × 10^{12} cm^{-2})	-	108 % (Δn = 0.38 × 10^{12} cm^{-2})

Additionally, the different carrier concentrations generated by doping could be related to potential carrier-carrier scattering effects within the MoS_2 channel. However, we expect such scattering effects would not be dominant (*66*). Therefore, we expect that the contribution from the carrier-carrier scattering effects would be relatively small.

In summary, in all the cases above, the mobility enhancement was significantly larger for the remotely doped devices than the directly doped devices. Therefore, the charged-impurity scattering was quantitatively proven to be suppressed by introducing the h-BN interlayer irrespective of the doping conditions used.

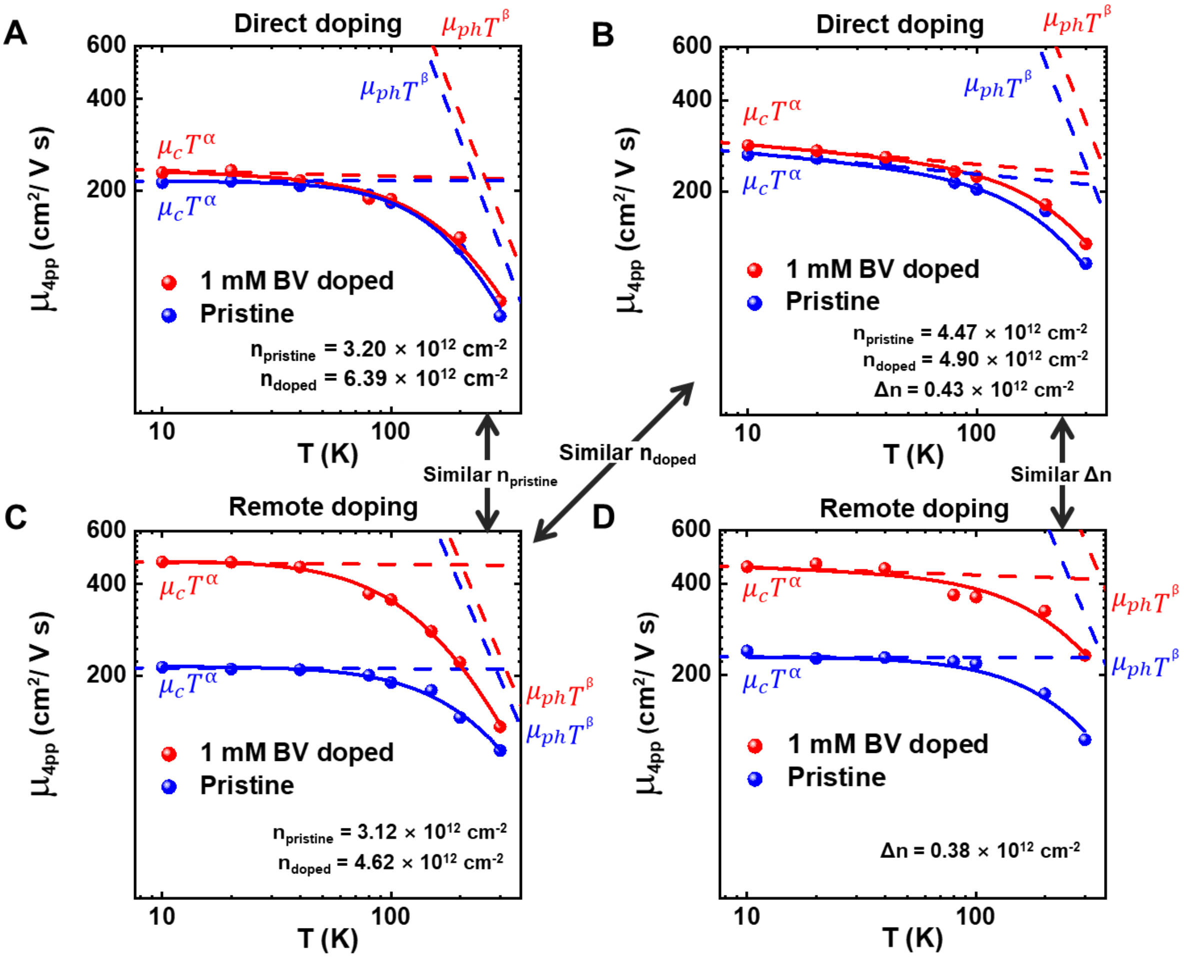

Fig. S16. Temperature-dependent mobility values for comparing the mobility enhancement after the doping for three cases: i) similar $n_{pristine}$, ii) similar n_{doped}, and iii) similar Δn, from 10 to 300 K. (A), (B) directly doped, **(C), (D)** remotely doped devices. The mobility values were extracted from the four-point probe conductance plots at V_{GS} = 80 V for pristine devices (blue lines) and doped devices (red lines).

Section S17. Comparison of directly and remotely doped devices under minimal screening from the gate-induced carriers

It is meaningful to compare mobilty values of the different devices when the screening effect of electrostatically accumulated carriers is absent in order to consider only the BV doped carriers. We resorted to comparing devices at $V_{GS} - V_{th}^{pristine} = 0\ V$ condition, which, we believe, is reasonable considering that different devices exhibit different $V_{th}^{pristine}$ (i.e. different $n_{pristine}$ and different electrostatically modulated carrier concentrations). The $V_{GS} - V_{th}^{pristine} = 0\ V$ condition means that the electrostatically modulated carrier concentration is kept minimal while the carrier concentration generated by the molecular doping remains (i.e. minimal screening from the electrostatically accumulated carriers).

As shown from our data in Fig. S17, we see a dramatic difference between the directly doped device and the remotely doped device (1 nm h-BN interlayer). While the $\mu_c T^{\alpha}$ of the directly doped device increased only by 6 %, the $\mu_c T^{\alpha}$ of the remotely doped device increased by 70 % after doping at $V_{GS} - V_{th}^{pristine} = 0\ V$ condition.

Therefore, we believe that our claim still remains valid when the screening effect of the electrostatically is kept minimal, which confirms that the effect of dopant-induced charged impurity scattering is likely to be dominant in our analysis.

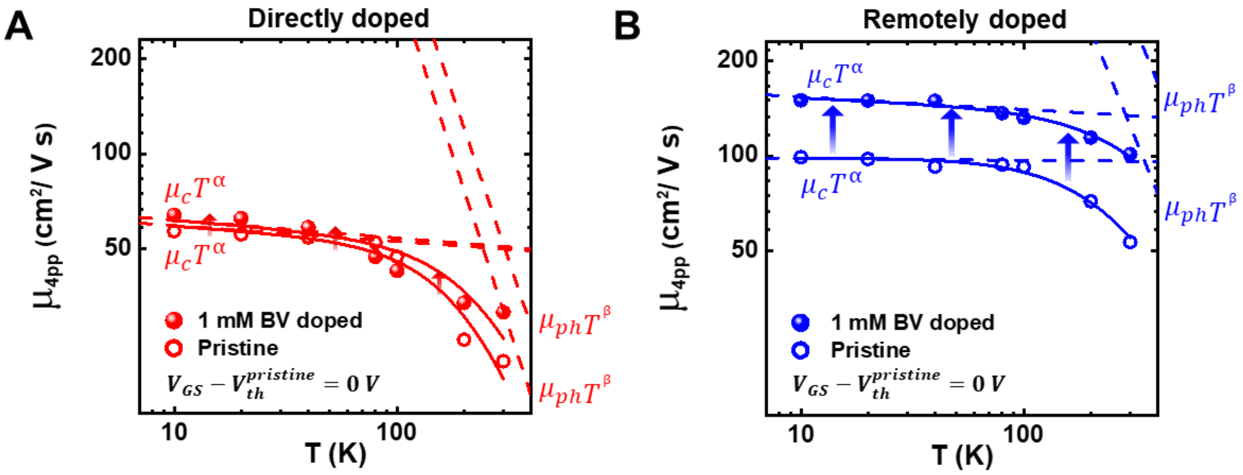

Fig. S17. Temperature-dependent mobility values. (**A**) directly doped and (**B**) remotely doped devices from 10 to 300 K. The mobility values were extracted from the four-point probe conductance plots at $V_{GS} - V_{th}^{pristine} = 0\ V$ condition which, is reasonable considering that different devices exhibit different $V_{th}^{pristine}$ for pristine devices (red open circle) and doped devices (filled circle).

Section S18. Theoretical models for calculating the mobility of directly doped and remotely doped MoS_2

Ong et al. reported a theoretical model for calculation of the charged impurity limited mobility of transition metal dichalcogenides (TMDCs) encapsulated by the different top and bottom dielectric materials (*58*). TMDC layer was assumed as a zero-thickness 2-dimensional electron gas (2DEG) in the model. The scattering potential of the single charged impurity in the MoS_2 channel ϕ_q^{scr} is given by $\phi_q^{\mathrm{scr}} = \frac{e^2 G_q}{\epsilon_{2D}(q,T)}$, where q is the wave vector, e is the elementary charge, and G_q is the Fourier transform of the Green's function solution of the Poisson equation. The G_q includes the electrostatic boundary conditions and is expressed as $G_q = [(\epsilon_{top} + \epsilon_{bottom} \coth(q t_{ox})] \, q]^{-1}$, where ϵ_{top} is the static permittivity of the top dielectric, ϵ_{bottom} is the static permittivity of the bottom dielectric and t_{ox} is the thickness of the bottom dielectric. In the direct doping case, the top dielectric is air $(\epsilon_{top} = 1)$ and the bottom dielectric is 270 nm-thick SiO_2 ($\epsilon_{bottom} = 3.9$ and t_{ox} = 270 nm). $\epsilon_{2D}(q,T)$ is the generalized static dielectric function expressed by $\epsilon_{2D}(q,T) = 1 - e^2 G_q \Pi(q,T,E_F)$, where $\Pi(q, T, E_F)$ is the static charge polarizability and E_F is the chemical potential. The static charge polarizability $\Pi(q, T, E_F)$ is expressed by $\Pi(q,T,E_F) = \int_0^{\infty} d\mu \frac{\Pi(q,0,\mu)}{4k_B T \; cosh^2(\frac{E_F-\mu}{2k_B T})}$, where

$\Pi(q,0,\mu) = \Pi(0,0,\mu)[1 - \Theta(q - 2k_F)\left\{1 - \left(\frac{2k_F}{q}\right)^2\right\}^{\frac{1}{2}}]$ with $k_F = \frac{\sqrt{2m_{eff}\mu}}{\hbar}$ and $\Pi(0,0,\mu) = -\frac{g m_{eff}}{(2\pi\hbar^2)}$. Here, we used an effective mass of electron 0.55 m_e for MoS_2 (*12*). The scattering rate for the single charged impurity in the MoS_2 channel (Γ_{imp}) can be given by the following equation

$$\Gamma_{\mathrm{imp}}(E_k) = \frac{1}{2\pi\hbar} \int d\mathbf{k}' \left|\phi_{|k-k'|}^{\mathrm{scr}}\right|^2 \times (1 - cos\theta_{\mathbf{kk}'})\delta(E_{\mathbf{k}} - E_{\mathbf{k}'}), \quad \text{(S1)}$$

where $\theta_{\mathbf{kk}'}$ is the scattering angle between the **k** and **k'** states. From Γ_{imp}, the charged impurity-limited mobility (μ_{imp}) is given by

$$\mu_{imp} = \frac{e}{\pi n \hbar^2 k_B T} \int_0^{\infty} f(E)[1 - f(E)]\,(n_{imp}\Gamma_{imp}(E))^{-1} E dE, \quad \text{(S2)}$$

where $f(E)$ is the Fermi-Dirac function, k_B is the Boltzmann constant, and n is the carrier density, and n_{imp} is the charged impurity density. By using this equation, the μ_{imp} of directly doped MoS_2 was calculated. Fig. S18A shows the μ_{imp} values of MoS_2 n_{imp} = 0.8, 1.0, 2.0 and 4×10^{12} cm^{-2}, and T = 10 K as a function of the carrier density of MoS_2.

In the remotely doped MoS_2 devices, the charged impurities generated by surface charge transfer doping are spatially separated by the h-BN spacer, unlike direct doping. To consider this spatial separation, we compare the Coulomb potential of charged impurity in the channel with spatially separated charged impurity with the following equation.

$$V^{direct}(\boldsymbol{r}) = \frac{e^2}{|\boldsymbol{r}|} = \int \frac{d^2\boldsymbol{q}}{(2\pi)^2} \frac{2\pi e^2}{|\boldsymbol{q}|} e^{i\boldsymbol{q}\cdot\boldsymbol{r}} \quad \text{(S3)}$$

$$V^{remote}(\boldsymbol{r}) = \frac{e^2}{\sqrt{r^2+d^2}} = \int \frac{d^2\boldsymbol{q}}{(2\pi)^2} \frac{2\pi e^2}{|\boldsymbol{q}|} e^{-|\boldsymbol{q}|d} e^{i\boldsymbol{q}\cdot\boldsymbol{r}}, \quad \text{(S4)}$$

where **r** is the position vector of electrons and d is the length of spatial separation. As we can see in the above equations, the $e^{-|\boldsymbol{q}|d}$ the term is added in spatially separated charged impurity case compared to that in the channel. Therefore, the scattering potential of spatially separated charged impurity is defined as $\phi_q^{\mathrm{remote}} = e^{-|\boldsymbol{q}|d}\phi_q^{\mathrm{scr}}$. By using this relation, the μ_{imp} of remotely doped MoS_2 devices was calculated. Fig. S18B shows the calculated μ_{imp} of remotely doped MoS_2 as a function of the carrier density of MoS_2 at T = 10 K. We plotted solid blue lines for d = 2 at n_{imp} = 1 and 4×10^{12} cm^{-2} and dashed red lines for d = 1 nm at n_{imp} = 1 and 4×10^{12} cm^{-2}.

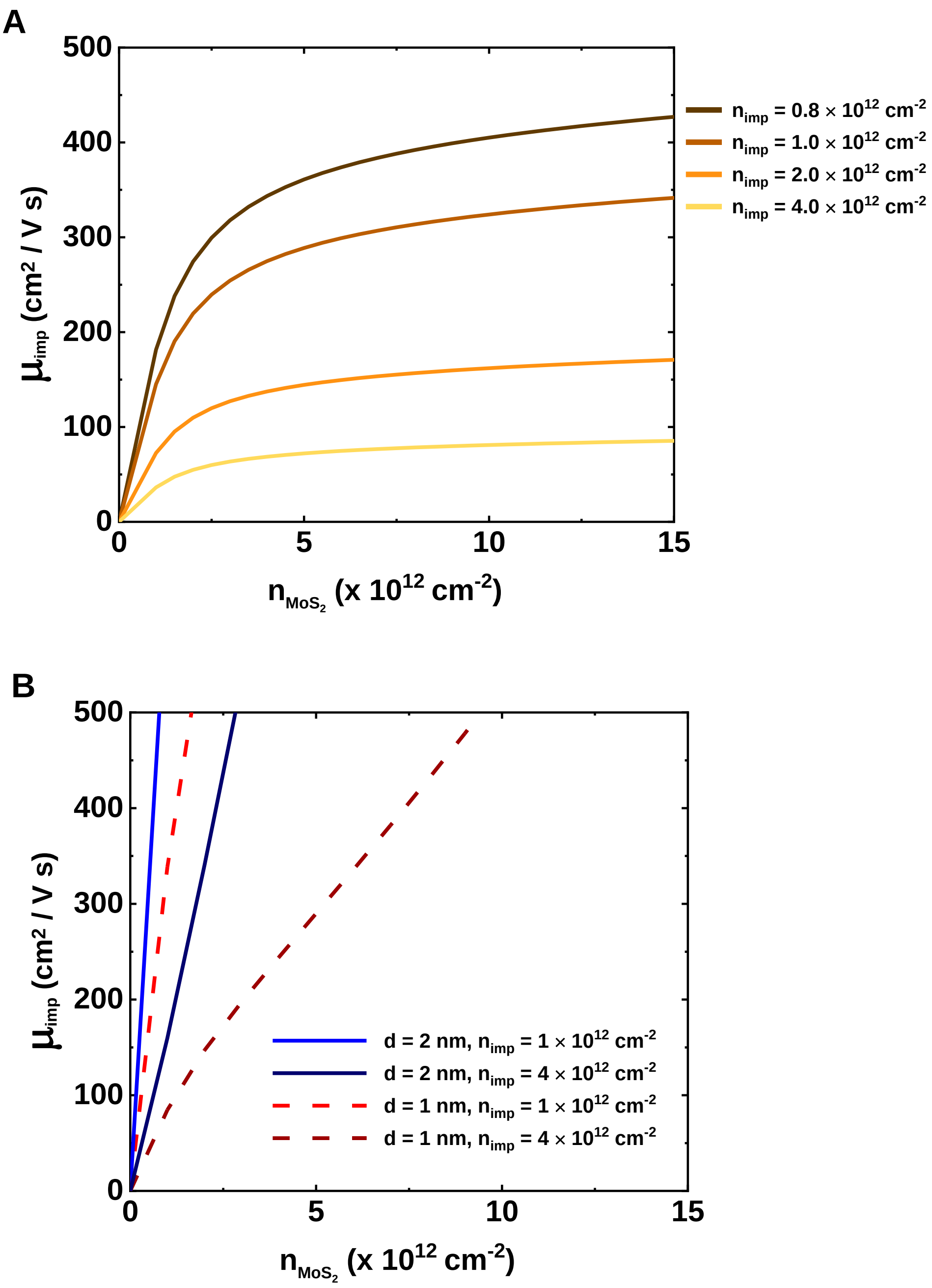

Fig. S18. Theoretical models for calculating the mobility of directly doped and remotely doped MoS_2. (**A**) The calculated μ_{imp} of directly doped MoS_2 as a function of the carrier density of MoS_2 at n_{imp} = 0.8, 1.0, 2.0 and 4.0 × 10^{12} cm^{-2} and T = 10 K. (**B**) The calculated μ_{imp} of remotely doped MoS_2 as a function of the carrier density of MoS_2 for d = 1 nm at n_{imp} = 1 and 4 × 10^{12} cm^{-2} and for d = 2 nm at n_{imp} = 1 and 4 × 10^{12} cm^{-2} and T = 10 K.

Section S19. Spatial inhomogeneity of the BV dopants on the MoS_2 surfaces

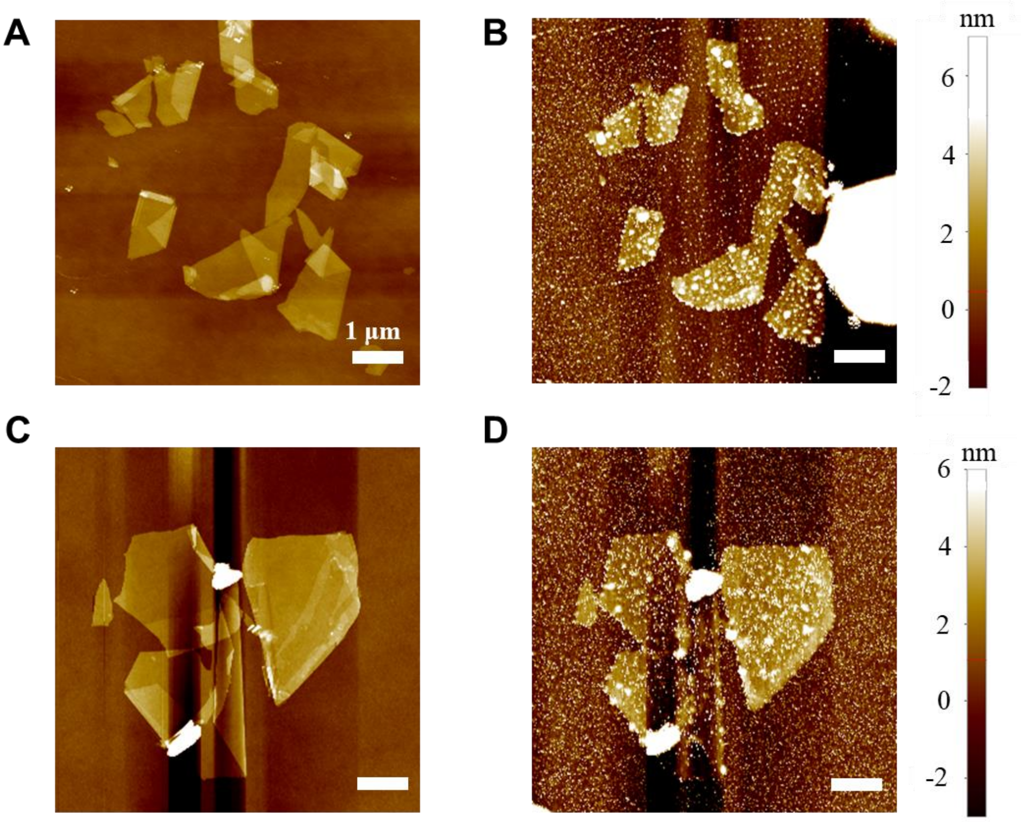

Fig. S19. AFM images of MoS_2 flakes. (**A, C**) undoped MoS_2 flakes and (**B, D**) BV-doped MoS_2 flakes with 1 mM concentration. In these AFM images, we could confirm that BV dopants (formed as clusters) were distributed non-uniformly on the MoS_2 surfaces, which supports the spatial inhomogeneity of the dopants. Such inhomogeneity of dopants can result in fluctuations of mobilities due to the charge scattering and screening effect.

REFERENCES AND NOTES

1. Q. H. Wang, K. Kalantar-Zadeh, A. Kis, J. N. Coleman, M. S. Strano, Electronics and optoelectronics of two-dimensional transition metal dichalcogenides. *Nat. Nanotechnol.* **7**, 699–712 (2012).

2. S. Manzeli, D. Ovchinnikov, D. Pasquier, O. V. Yazyev, A. Kis, 2D transition metal dichalcogenides. *Nat. Rev. Mater.* **2**, 17033 (2017).

3. K. F. Mak, C. Lee, J. Hone, J. Shan, T. F. Heinz, Atomically thin MoS_2: A new direct-gap semiconduc tor. *Phys. Rev. Lett.* **105**, 136805 (2010).

4. Y. L. Huang, Y. Chen, W. Zhang, S. Y. Quek, C.-H. Chen, L.-J. Li, W.-T. Hsu, W.-H. Chang, Y. J. Z heng, W. Chen, A. T. S. Wee, Bandgap tunability at single-layer molybdenum disulphide grain boun daries. *Nat. Commun.* **6**, 6298 (2015).

5. W. Zhang, Q. Wang, Y. Chen, Z. Wang, A. T. S. Wee, Van der Waals stacked 2D layered materials fo r optoelectronics. *2D Mater.* **3**, 022001 (2016).

6. C.-H. Lee, G.-H. Lee, A. M. van der Zande, W. Chen, Y. Li, M. Han, X. Cui, G. Arefe, C. Nuckolls, T. F. Heinz, J. Guo, J. Hone, P. Kim, Atomically thin p–n junctions with van der Waals heterointerfa ces. *Nat. Nanotechnol.* **9**, 676–681 (2014).

7. M. E. Beck, M. C. Hersam, Emerging opportunities for electrostatic control in atomically thin device s. *ACS Nano* **14**, 6498–6518 (2020).

8. J. Xu, L. Chen, Y.-W. Dai, Q. Cao, Q.-Q. Sun, S.-J. Ding, H. Zhu, D. W. Zhang, A two-dimensional s emiconductor transistor with boosted gate control and sensing ability. *Sci. Adv.* **3**, e1602246 (2017).

9. D.-H. Lien, S. Z. Uddin, M. Yeh, M. Amani, H. Kim, J. W. Ager III, E. Yablonovitch, A. Javey, Elect rical suppression of all nonradiative recombination pathways in monolayer semiconductors. *Science* **364**, 468–471 (2019).

10. H. Kim, S. Z. Uddin, N. Higashitarumizu, E. Rabani, A. Javey, Inhibited nonradiative decay at all ex citon densities in monolayer semiconductors. *Science* **373**, 448–452 (2021).

11. J. Shim, S.-H. Bae, W. Kong, D. Lee, K. Qiao, D. Nezich, J. Park Yong, R. Zhao, S. Sundaram, X. L i, H. Yeon, C. Choi, H. Kum, R. Yue, G. Zhou, Y. Ou, K. Lee, J. Moodera, X. Zhao, J.-H. Ahn, C. H inkle, A. Ougazzaden, J. Kim, Controlled crack propagation for atomic precision handling of wafer-s cale two-dimensional materials. *Science* **362**, 665–670 (2018).

12. S. B. Desai, S. R. Madhvapathy, A. B. Sachid, J. P. Llinas, Q. Wang, G. H. Ahn, G. Pitner, M. J. Ki m, J. Bokor, C. Hu, H.-S. P. Wong, A. Javey, MoS_2 transistors with 1-nanometer gate lengths. *Scien ce* **354**, 99–102 (2016).

13. B. Radisavljevic, A. Radenovic, J. Brivio, V. Giacometti, A. Kis, Single-layer MoS_2 transistors. *Nat. Nanotechnol.* **6**, 147–150 (2011).

14. J. G. Roch, G. Froehlicher, N. Leisgang, P. Makk, K. Watanabe, T. Taniguchi, R. J. Warburton, Spin-polarized electrons in monolayer MoS_2. *Nat. Nanotechnol.* **14**, 432–436 (2019).

15. K. F. Mak, K. L. McGill, J. Park, P. L. McEuen, The valley hall effect in MoS_2 transistors. *Science* **344**, 1489–1492 (2014).

16. J. M. Lu, O. Zheliuk, I. Leermakers, N. F. Q. Yuan, U. Zeitler, K. T. Law, J. T. Ye, Evidence for two-dimensional ising superconductivity in gated MoS_2. *Science* **350**, 1353–1357 (2015).

17. Y. Saito, Y. Nakamura, M. S. Bahramy, Y. Kohama, J. Ye, Y. Kasahara, Y. Nakagawa, M. Onga, M. Tokunaga, T. Nojima, Y. Yanase, Y. Iwasa, Superconductivity protected by spin–valley locking in ion-gated MoS_2. *Nat. Phys.* **12**, 144–149 (2016).

18. X. Liu, M. C. Hersam, 2D materials for quantum information science. *Nat. Rev. Mater.* **4**, 669–684 (2019).

19. A. Aljarb, J.-H. Fu, C.-C. Hsu, C.-P. Chuu, Y. Wan, M. Hakami, D. R. Naphade, E. Yengel, C.-J. Lee, S. Brems, T.-A. Chen, M.-Y. Li, S.-H. Bae, W.-T. Hsu, Z. Cao, R. Albaridy, S. Lopatin, W.-H. Chang, T. D. Anthopoulos, J. Kim, L.-J. Li, V. Tung, Ledge-directed epitaxy of continuously self-aligned single-crystalline nanoribbons of transition metal dichalcogenides. *Nat. Mater.* **19**, 1300–1306 (2020).

20. D. Kiriya, M. Tosun, P. Zhao, J. S. Kang, A. Javey, Air-stable surface charge transfer doping of MoS_2 by benzyl viologen. *J. Am. Chem. Soc.* **136**, 7853–7856 (2014).

21. B. Radisavljevic, A. Kis, Mobility engineering and a metal–insulator transition in monolayer MoS_2. *Nat. Mater.* **12**, 815–820 (2013).

22. S. Mouri, Y. Miyauchi, K. Matsuda, Tunable photoluminescence of monolayer MoS_2 via chemical doping. *Nano Lett.* **13**, 5944–5948 (2013).

23. L. Yang, K. Majumdar, H. Liu, Y. Du, H. Wu, M. Hatzistergos, P. Y. Hung, R. Tieckelmann, W. Tsai, C. Hobbs, P. D. Ye, Chloride molecular doping technique on 2D materials: WS_2 and MoS_2. *Nano Lett.* **14**, 6275–6280 (2014).

24. H. Fang, S. Chuang, T. C. Chang, K. Takei, T. Takahashi, A. Javey, High-performance single layered WSe_2 p-FETs with chemically doped contacts. *Nano Lett.* **12**, 3788–3792 (2012).

25. H. Fang, M. Tosun, G. Seol, T. C. Chang, K. Takei, J. Guo, A. Javey, Degenerate n-doping of few-layer transition metal dichalcogenides by potassium. *Nano Lett.* **13**, 1991–1995 (2013).

26. S. Zhang, H. M. Hill, K. Moudgil, C. A. Richter, A. R. H. Walker, S. Barlow, S. R. Marder, C. A. Hacker, S. J. Pookpanratana, Controllable, wide-ranging n-doping and p-doping of monolayer group 6 transition-metal disulfides and diselenides. *Adv. Mater.* **30**, 1802991 (2018).

27. M.-Y. Tsai, S. Zhang, P. M. Campbell, R. R. Dasari, X. Ba, A. Tarasov, S. Graham, S. Barlow, S. R.

Marder, E. M. Vogel, Solution-processed doping of trilayer WSe_2 with redox-active molecules. *Chem. Mater.* **29**, 7296–7304 (2017).

28. S. A. Paniagua, J. Baltazar, H. Sojoudi, S. K. Mohapatra, S. Zhang, C. L. Henderson, S. Graham, S. Barlow, S. R. Marder, Production of heavily n- and p-doped CVD graphene with solution-processed redox-active metal–organic species. *Mater. Horiz.* **1**, 111–115 (2014).

29. H. Gao, J. Suh, M. C. Cao, A. Y. Joe, F. Mujid, K.-H. Lee, S. Xie, P. Poddar, J.-U. Lee, K. Kang, P. Kim, D. A. Muller, J. Park, Tuning electrical conductance of MoS_2 Monolayers through substitutional doping. *Nano Lett.* **20**, 4095–4101 (2020).

30. T. Zhang, K. Fujisawa, F. Zhang, M. Liu, M. C. Lucking, R. N. Gontijo, Y. Lei, H. Liu, K. Crust, T. Granzier-Nakajima, H. Terrones, A. L. Elías, M. Terrones, Universal in situ substitutional doping of transition metal dichalcogenides by liquid-phase precursor-assisted synthesis. *ACS Nano* **14**, 4326–4335 (2020).

31. S. K. Pandey, H. Alsalman, J. G. Azadani, N. Izquierdo, T. Low, S. A. Campbell, Controlled p-type substitutional doping in large-area monolayer WSe_2crystals grown by chemical vapor deposition. *Nanoscale* **10**, 21374–21385 (2018).

32. V. Iberi, L. Liang, A. V. Ievlev, M. G. Stanford, M.-W. Lin, X. Li, M. Mahjouri-Samani, S. Jesse, B. G. Sumpter, S. V. Kalinin, D. C. Joy, K. Xiao, A. Belianinov, O. S. Ovchinnikova, Nanoforging single layer $MoSe_2$ through defect engineering with focused helium ion beams. *Sci. Rep.* **6**, 30481 (2016).

33. K. Xu, Y. Zhao, Z. Lin, Y. Long, Y. Wang, M. Chan, Y. Chai, Doping of two-dimensional MoS_2 by high energy ion implantation. *Semicond. Sci. Technol.* **32**, 124002 (2017).

34. D. Wang, X.-B. Li, H.-B. Sun, Modulation doping: A strategy for 2D materials electronics. *Nano Lett.* **21**, 6298–6303 (2021).

35. D. Lee, J. J. Lee, Y. S. Kim, Y. H. Kim, J. C. Kim, W. Huh, J. Lee, S. Park, H. Y. Jeong, Y. D. Kim, C.-H. Lee, Remote modulation doping in van der Waals heterostructure transistors. *Nat. Electron.* **4**, 664–670 (2021).

36. K. Kang, S. Watanabe, K. Broch, A. Sepe, A. Brown, I. Nasrallah, M. Nikolka, Z. Fei, M. Heeney, D. Matsumoto, K. Marumoto, H. Tanaka, S.-i. Kuroda, H. Sirringhaus, 2D coherent charge transport in highly ordered conducting polymers doped by solid state diffusion. *Nat. Mater.* **15**, 896–902 (2016).

37. E. F. Schubert, Doping in heterostructures, quantum wells, and superlattices, in *Doping in III-V Semiconductors* (Cambridge Univ. Press, 1993), pp. 392–397.

38. H. L. Störmer, The fractional quantum hall effect, in *Advances in Solid State Physics* (Springer Berlin Heidelberg, 1984), vol. 24, pp. 25–44.

39. G. Springholz, G. Ihninger, G. Bauer, M. M. Olver, J. Z. Pastalan, S. Romaine, B. B. Goldberg, Mod

ulation doping and observation of the integral quantum Hall effect in $PbTe/Pb_{1-x}Eu_xTe$ multiquantu m wells. *Appl. Phys. Lett.* **63**, 2908–2910 (1993).

40. J.-K. Kim, K. Cho, J. Jang, K.-Y. Baek, J. Kim, J. Seo, M. Song, J. Shin, J. Kim, S. S. P. Parkin, J.-H. Lee, K. Kang, T. Lee, Molecular dopant-dependent charge transport in surface-charge-transfer-dope d tungsten diselenide field effect transistors. *Adv. Mater.* **33**, 2101598 (2021).

41. S.-L. Li, K. Komatsu, S. Nakaharai, Y.-F. Lin, M. Yamamoto, X. Duan, K. Tsukagoshi, Thickness s caling effect on interfacial barrier and electrical contact to two-dimensional MoS_2 layers. *ACS Nano* **8**, 12836–12842 (2014).

42. B. Chamlagain, S. S. Withanage, A. C. Johnston, S. I. Khondaker, Scalable lateral heterojunction by chemical doping of 2D TMD thin films. *Sci. Rep.* **10**, 12970 (2020).

43. C. Gong, H. Zhang, W. Wang, L. Colombo, R. M. Wallace, K. Cho, Band alignment of two-dimensi onal transition metal dichalcogenides: Application in tunnel field effect transistors. *Appl. Phys. Lett.* **103**, 053513 (2013).

44. F. Zhao, Q. Li, K. Han, T. Lian, Mechanism of efficient viologen radical generation by ultrafast elect ron transfer from cds quantum dots. *J. Phys. Chem. C* **122**, 17136–17142 (2018).

45. J. Wang, Q. Yao, C.-W. Huang, X. Zou, L. Liao, S. Chen, Z. Fan, K. Zhang, W. Wu, X. Xiao, C. Jia ng, W.-W. Wu, High mobility MoS_2 transistor with low schottky barrier contact by using atomic thic k h-BN as a tunneling layer. *Adv. Mater.* **28**, 8302–8308 (2016).

46. Y. Liu, H. Wu, H.-C. Cheng, S. Yang, E. Zhu, Q. He, M. Ding, D. Li, J. Guo, N. O. Weiss, Y. Huan g, X. Duan, Toward barrier free contact to molybdenum disulfide using graphene electrodes. *Nano L ett.* **15**, 3030–3034 (2015).

47. A. Allain, A. Kis, Electron and hole mobilities in single-layer WSe_2. *ACS Nano* **8**, 7180–7185 (2014).

48. X. Cui, G.-H. Lee, Y. D. Kim, G. Arefe, P. Y. Huang, C.-H. Lee, D. A. Chenet, X. Zhang, L. Wang, F. Ye, F. Pizzocchero, B. S. Jessen, K. Watanabe, T. Taniguchi, D. A. Muller, T. Low, P. Kim, J. Ho ne, Multi-terminal transport measurements of MoS_2 using a van der Waals heterostructure device pla tform. *Nat. Nanotechnol.* **10**, 534–540 (2015).

49. S.-L. Li, K. Wakabayashi, Y. Xu, S. Nakaharai, K. Komatsu, W.-W. Li, Y.-F. Lin, A. Aparecido-Fer reira, K. Tsukagoshi, Thickness-dependent interfacial Coulomb scattering in atomically thin field-eff ect transistors. *Nano Lett.* **13**, 3546–3552 (2013).

50. N. R. Pradhan, D. Rhodes, S. Memaran, J. M. Poumirol, D. Smirnov, S. Talapatra, S. Feng, N. Perea -Lopez, A. L. Elias, M. Terrones, P. M. Ajayan, L. Balicas, Hall and field-effect mobilities in few la yered p-WSe_2 field-effect transistors. *Sci. Rep.* **5**, 8979 (2015).

51. N. Ma, D. Jena, Charge scattering and mobility in atomically thin semiconductors. *Phy. Rev. X* **4**, 01 1043 (2014).

52. A. T. Neal, H. Liu, J. Gu, P. D. Ye, Magneto-transport in MoS_2: Phase coherence, spin–orbit scatteri ng, and the hall factor. *ACS Nano* **7**, 7077–7082 (2013).

53. B. W. H. Baugher, H. O. H. Churchill, Y. Yang, P. Jarillo-Herrero, Intrinsic electronic transport prop erties of high-quality monolayer and bilayer MoS_2. *Nano Lett.* **13**, 4212–4216 (2013).

54. K. Kaasbjerg, K. S. Thygesen, K. W. Jacobsen, Phonon-limited mobility in *n*-type single-layer MoS_2 from first principles. *Phys. Rev. B* **85**, 115317 (2012).

55. A. Rai, A. Valsaraj, H. C. Movva, A. Roy, R. Ghosh, S. Sonde, S. Kang, J. Chang, T. Trivedi, R. De y, S. Guchhait, S. Larentis, L. F. Register, E. Tutuc, S. K. Banerjee, Air stable doping and intrinsic m obility enhancement in monolayer molybdenum disulfide by amorphous titanium suboxide encapsula tion. *Nano Lett.* **15**, 4329–4336 (2015).

56. H. G. Ji, P. Solís-Fernández, D. Yoshimura, M. Maruyama, T. Endo, Y. Miyata, S. Okada, H. Ago, C hemically tuned p- and n-type WSe_2 monolayers with high carrier mobility for advanced electronics. *Adv. Mater.* **31**, 1903613 (2019).

57. D. Lembke, A. Allain, A. Kis, Thickness-dependent mobility in two-dimensional MoS_2 transistors. *Nanoscale* **7**, 6255–6260 (2015).

58. Z.-Y. Ong, M. V. Fischetti, Mobility enhancement and temperature dependence in top-gated single-l ayer MoS_2. *Phys. Rev. B* **88**, 165316 (2013).

59. X. Liu, Z. Wang, K. Watanabe, T. Taniguchi, O. Vafek, J. I. A. Li, Tuning electron correlation in ma gic-angle twisted bilayer graphene using Coulomb screening. *Science* **371**, 1261–1265 (2021).

60. M. Yarali, Y. Zhong, S. N. Reed, J. Wang, K. A. Ulman, D. J. Charboneau, J. B. Curley, D. J. Hyne k, J. V. Pondick, S. Yazdani, N. Hazari, S. Y. Quek, H. Wang, J. J. Cha, Near-unity molecular dopin g efficiency in monolayer MoS_2. *Adv. Electron. Mater.* **7**, 2000873 (2021).

61. D. G. Purdie, N. M. Pugno, T. Taniguchi, K. Watanabe, A. C. Ferrari, A. Lombardo, Cleaning interfa ces in layered materials heterostructures. *Nat. Commun.* **9**, 5387 (2018).

62. S. M. Kim, J. H. Jang, K. K. Kim, H. K. Park, J. J. Bae, W. J. Yu, I. H. Lee, G. Kim, D. D. Loc, U. J. Kim, E.-H. Lee, H.-J. Shin, J.-Y. Choi, Y. H. Lee, Reduction-controlled viologen in bisolvent as an environmentally stable n-type dopant for carbon nanotubes. *J. Am. Chem. Soc.* **131**, 327–331 (2009).

63. W. Park, J. Park, J. Jang, H. Lee, H. Jeong, K. Cho, S. Hong, T. Lee, Oxygen environmental and pas sivation effects on molybdenum disulfide field effect transistors. *Nanotechnology* **24**, 095202 (201 3).

64. K. Cho, W. Park, J. Park, H. Jeong, J. Jang, T.-Y. Kim, W. K. Hong, S. Hong, T. Lee, Electric stress -induced threshold voltage instability of multilayer MoS_2 field effect transistors. *ACS Nano* **7**, 7751–7758 (2013).

65. J. G. Simmons, Generalized formula for the electric tunnel effect between similar electrodes separate d by a thin insulating film. *J. Appl. Phys.* **34**, 1793–1803 (1963)

66. S.-L. Li, K. Tsukagoshi, E. Orgiu, P. Samori, Charge transport and mobility engineering in two-dime nsional transition metal chalcogenide semiconductors. *Chem. Soc. Rev.* **45**, 118–151 (2016)
